# *NF2* is Essential for Human Endoderm Development

**DOI:** 10.1002/advs.202410909

**Published:** 2025-02-08

**Authors:** Minjin Jeong, Dongjun Han, Preetida Bhetariya, D. Bradley Welling, Miodrag Stojkovic, Konstantina M. Stankovic

**Affiliations:** ^1^ Department of Otolaryngology‐Head and Neck Surgery Stanford University School of Medicine Stanford CA 94305 USA; ^2^ Department of Otolaryngology‐Head and Neck Surgery Massachusetts Eye and Ear and Harvard Medical School Boston MA 02114 USA; ^3^ Bioinformatics Core Harvard T.H. Chan School of Public Health Boston MA 02115 USA; ^4^ Department of Neurosurgery Stanford University School of Medicine Stanford CA 94304 USA; ^5^ Wu Tsai Neurosciences Institute Stanford University Stanford CA 94305 USA

**Keywords:** endoderm, hippo pathway, human induced pluripotent stem cells, *NF2*, YAP1

## Abstract

Vertebrate embryogenesis requires the precisely timed specification of 3 germ cell layers— ectoderm, mesoderm, and endoderm— which give rise to tissues and organs in the developing organism. The tumor suppressor gene NF2, moesin‐ezrin‐radixin like (MERLIN) tumor suppressor (Nf2) is expressed in all 3 germ layers during mouse development and its homozygous deletion causes embryonic lethality. People with heterozygous NF2 mutations typically develop Schwann cell tumors, especially vestibular schwannoma, but the specific role of NF2 in human embryonic development is unclear. Here, human induced pluripotent stem cells (hiPSCs) are used to demonstrate that NF2 is essential for endoderm specification and formation in humans. Although endoderm differentiation is not impaired in hiPSCs with heterozygous NF2 mutation, NF2 knockout (NF2^−/−^) abolished the capacity to form endoderm in vitro, confirmed by loss of expression of endoderm‐related genes and proteins, or teratomas in vivo. This defect is mediated by the nuclear translocation of yes‐associated protein 1 (YAP1), a transcription co‐activator regulating lineage fate via the Hippo pathway and subsequent YAP1‐mediated shutdown of Activin/Nodal signaling. Endoderm formation can be rescued via YAP1 knockdown or forced re‐expression of NF2 in NF2^−/−^ cells. Taken together, the essential role of NF2 during endoderm specification in human embryogenesis as a regulator of YAP1 is reported.

## Graphical abstract

This study demonstrates that NF2, moesin‐ezrin‐radixin like (MERLIN) tumor suppressor (NF2) is essential for human endoderm formation. NF2 knockout human induced pluripotent stem cells fail to form endoderm both in vitro and in vivo due to yes‐associated protein 1 (YAP1) nuclear translocation, redirecting differentiation toward myofibroblast‐like cells. This defect is reversed by YAP1 knockdown or forced NF2 expression, underscoring NF2's pivotal role in lineage fate determination.

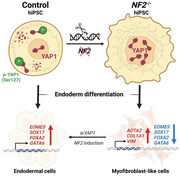

## 1 Introduction

Embryonic development is a complex process regulated by a variety of signaling pathways, including Notch, transforming growth factor β (TGFβ), WNT/β ‐catenin, Hippo, receptor tyrosine kinase, and Hedgehog.^[^ ^1^ ^]^ The protein merlin, encoded by the NF2, moesin‐ezrin‐radixin like (MERLIN) tumor suppressor (*Nf2*) gene in mice, is essential to segregate the inner cell mass from the trophectoderm in preimplantation embryo,^[^ ^2^ ^]^ and helps orchestrate these pathways to ensure normal embryonic development, including the formation of germ layers and timing of cell pluripotency and proliferation.^[^ ^3^ ^]^ Accordingly, loss of *Nf2* function leads to mouse embryonic lethality due to a collapsed extraembryonic region or failure to initiate gastrulation, indicated by absent expression of primitive streak (T‐box transcription factor T, *TBXT*) and definitive endoderm (Forkhead box protein A2, *FOXA2)* markers.^[^ ^3c^ ^]^ Mouse embryonic stem cells with homozygous loss of *Nf2* primarily develop into extraembryonic mesoderm in chimeric embryos, with some mutant cells contributing to all 3 embryonic germ layers.^[^ ^3c^ ^]^

Nf2 (human homolog NF2) is an upstream regulator of the Hippo pathway, which controls organ size through the regulation of cell proliferation and apoptosis.^[^ ^4^ ^]^ In mammals, the Hippo pathway is composed of several key components, including mammalian STE20‐like kinase1/2, salvador family WW domain containing protein 1, MOB kinase activator 1A/B, large tumor suppressor kinase1/2, yes‐associated protein 1 (YAP1), WW domain containing transcription regulator 1 (TAZ), and the transcriptional enhanced associate domain (TEAD) family.^[^ ^5^ ^]^ Prior studies, primarily in mouse embryonic stem cells or mouse models, have established the important roles of the Hippo pathway and its effectors in self‐renewal and differentiation in vitro^[^ ^6^ ^]^ (Table S1A, Supplementary Table) and mammalian development in vivo^[^ ^3^, ^7^ ^]^ (Table S1B, Supplementary Table). The consequences of removing each component of the Hippo pathway have ranged from impaired differentiation of all 3 germ layers and embryonic lethality to a completely normal phenotype.^[^ ^3^, ^6^ ^]^

There is comparatively little evidence on the roles of the Hippo pathway in human embryonic development and early germ layer differentiation. Although models targeting *YAP1* ^[^ ^8^ ^]^ and *TAZ*,^[^ ^9^ ^]^ major downstream effectors of the Hippo pathway, in human pluripotent stem cells have suggested that the governance of *YAP1* activity is essential for lineage differentiation,^[^ ^8^ ^]^ the lack of appropriate human developmental models has hindered a comprehensive understanding of other Hippo pathway effectors, including that of human homolog *NF2* (Table S1C, Supplementary Table). Furthermore, while humans with heterozygous *NF2* mutations develop schwannomas associated with the inherited disorder neurofibromatosis type 2, *Nf2* hemizygous mice do not exhibit the same phenotype^[^ ^10^ ^]^ (Table S1D, Supplementary Table), making them an inaccurate model for studying human *NF2* mutant phenotypes. These observations underscore the need to develop in vitro models for studying human *NF2* to better understand its role in human development.

Here, we generated *NF2* knockout (*NF2^−/−^*) human induced pluripotent stem cell (hiPSC) lines utilizing clustered regularly interspaced short palindromic repeats and CRISPR‐associated protein 9 (CRISPR/Cas9) technology and investigated their ability to differentiate into all 3 germ layers in vitro and in vivo. The loss of *NF2* prevented YAP1 phosphorylation and degradation, leading to its nuclear expression, which in turn blocked endoderm differentiation by inactivating the Activin/Nodal pathway. Simultaneously, the absence of *NF2* promoted a myoblast‐like fate through the activation of TGFβ signaling. Additionally, we confirmed that *NF2^−/−^* hiPSCs exhibited a diminished capacity to form teratomas, particularly affecting endoderm lineage in vivo. These phenotypes were rescued by *YAP1* knockdown or by doxycycline (Dox)‐inducible *NF2* overexpression in *NF2^−/−^* cells. Our findings suggest that NF2‐mediated regulation of YAP1 is crucial for controlling endoderm differentiation during early germ layer specification in humans.

## 2 Results

### 2.1 *NF2* is Essential for Endoderm Formation In Vitro and In Vivo

**Figure 1:**
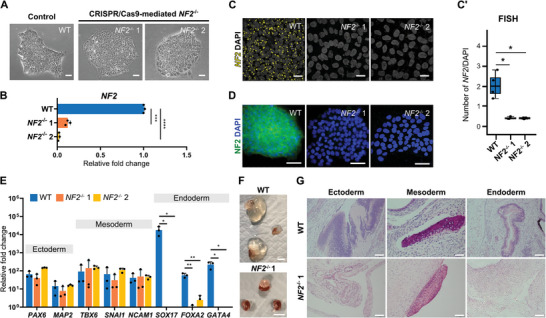
Loss of *NF2* profoundly impairs endoderm formation in vitro and in vivo. A) Representative phase contrast images of WT, and CRISPR/Cas9‐mediated *NF2^−/−^* 1 and *NF2^−/−^* 2 hiPSC lines. Scale bars, 50 µm. B) Average fold‐change of *NF2* mRNA expression in hiPSCs. The average expression of *NF2* in WT was set to 1. Both *NF2*‐deficient hiPSC lines had significantly lower *NF2* expression compared to WT. C) Fluorescence images and quantification (C’) of *NF2* RNA via fluorescent in situ hybridization in hiPSCs. Scale bars, 20 µm. D) NF2 protein expression detected by immunocytochemistry. Scale bars, 20 µm. E) Tri‐lineage differentiation as confirmed by markers of ectoderm (*PAX6*, *MAP2*), mesoderm (*TBX6*, *SNAI1*, *NCAM1*), and endoderm (*SOX17*, *FOXA2*, *GATA4*). Relative fold‐change in mRNA expression in differentiated cells from hiPSCs demonstrates, absent or low levels of endoderm markers in *NF2^−/−^* cells. The average expression in WT hiPSCs was set to 1. F) Three large renal teratomas developed in mice injected with WT hiPSCs, compared with small teratomas after injection with *NF2^−/−^* 1 hiPSCs. Scale bars, 10 µm. G) Histology of the renal teratomas stained with hematoxylin and eosin demonstrates that loss of *NF2* blocks endoderm but not ectoderm or mesoderm formation in vivo. Scale bars, 100 µm. ^*^ *p *< 0.05, ^**^ *p* < 0.01, ^***^ *p* < 0.001, ^****^ *p* < 0.0001. See “Quantification and Statistical Analysis” in the Experimental Section for statistics and experimental information.

The *NF2* exon 2–4 region spans the most frequently mutated sites in patients with *NF2*‐related schwannomatosis (previously known as neurofibromatosis type 2)^[^ ^11^ ^]^ and deletion of exon 2–3 causes complete loss of *Nf2* function and failure of embryogenesis in mice.^[^ ^3^, ^10^ ^]^ To examine the role of *NF2* during early lineage commitment in hiPSC, we generated *NF2* exon 2–4 targeted knockout (*NF2* ^−/−^) clones from a control wildtype (WT) hiPSC using CRISPR/Cas9. Two independent *NF2* knockout clones (*NF2* ^−/−^ 1 and *NF2* ^−/−^ 2) were generated and biallelic *NF2* exon 2–4 excision was verified by genomic DNA sequencing (**Figure** **1**A; Figure S1A–F, Supporting Information). The *NF2* ^−/−^ hiPSC lines were further validated with karyotyping (Figure S1B,E, Supporting Information) and immunocytochemistry for pluripotency markers (Figure S1C,F, Supporting Information).

Next, *NF2* expression levels at both the pre‐ and post‐translational stages were assessed in hiPSCs. Using primers binding to exons 2–3, *NF2* messenger RNA expression was found to be reduced by 87.88% and 98.00% in the *NF2^−/−^* 1 and *NF2^−/−^* 2 hiPSCs, respectively, compared to WT (Figure 1B). Visualization and quantification of *NF2* RNA by fluorescent in situ hybridization (FISH), using an oligo binding site on exon 3, revealed that *NF2* RNA per cell was completely absent in *NF2^−/−^* 1 and *NF2^−/−^* 2 hiPSCs (Figure 1C,C’). Immunocytochemistry additionally confirmed the absence of NF2 protein in *NF2^−/−^* 1 and *NF2^−/−^* 2 hiPSCs (Figure 1D).

In addition, *NF2* ^−/−^ 1, *NF2* ^−/−^ 2 and isogenic control WT were individually tested for their ability to differentiate into all 3 germ layers in vitro via a monolayer differentiation method using a validated commercial kit. Notably, *NF2^−/−^* 1 and *NF2^−/−^* 2 hiPSCs did not give rise to endoderm‐related cells, as evidenced by the complete absence or low levels of expression of the endoderm lineage‐specific markers (*SOX17*, *FOXA2*, and *GATA4)*, whereas the formation of ectoderm and mesoderm, and genes related to their specification, were unaffected (Figure 1E). In contrast, a patient‐derived line containing heterozygous mutation of *NF2* called *NF2^−/+^* (sex‐ and age‐matched from the WT, *NF2^−/−^* 1 and *NF2^−/−^* 2; Figure S1G–I, Supporting Information) did not prevent tri‐lineage differentiation (Figure S1J, Supporting Information). In *NF2^−/+^* hiPSCs, the heterozygous deletion of “G” in a splicing donor site of intron (NC_000022.11:g.29668447delG) next to exon 10 was confirmed by single nucleotide polymorphisms genotyping mutation analysis. *NF2^−/+^* hiPSCs had a single mutation in one allele of the *NF2* intron which may be compensated for by the normal *NF2* allele. These results suggest that *NF2*, especially exon 2–4, is crucial for endoderm formation via regulation of *SOX17, FOXA2, and GATA4*, and that one functional *NF2* allele could be sufficient to maintain normal lineage specification.

Then, the WT and *NF2* ^−/−^ 1 hiPSCs were injected into severe combined immunodeficiency mice for an in vivo teratoma formation assay (Figure 1F; Figure S1K, Supporting Information).^[^ ^12^ ^]^ On average, mice receiving WT hiPSCs developed significantly larger renal teratomas than those receiving *NF2* ^−/−^ 1 hiPSCs (*n* = 3, mean± standard deviation [SD] area: 1967.7 ± 1276.5 vs 45.5 ± 40.2 mm^2^; ^*^ *p *< 0.05) (Figure S1L, Supporting Information). Histological analysis of the teratomas showed that both WT and *NF2* ^−/−^ 1 hiPSCs had differentiated into tissues derived from ectoderm (i.e., neural or skin epidermal tissue) and mesoderm (i.e., cartilage) (Figure 1G). However, glandular gut‐like epithelium, derived from endoderm, was observed only in WT hiPSC‐derived teratomas (total 5 sections, average size: 625 µm). In summary, *NF2* expression is essential for endoderm development in vitro and in vivo.

### 2.2 *NF2* Deletion Results in Dysregulation of Cytoskeleton Organization

Early lineage specification relies on tightly controlled, consecutive changes in developmental gene expression that commence from the pluripotent stem cells,^[^ ^13^ ^]^ primarily governed by pluripotency markers or key transcription factors such as *OCT4*, *NANOG*, and *SOX2*.^[^ ^14^ ^]^ To determine whether *NF2* deletion blocked endoderm formation by preventing the expression of genes maintaining the pluripotent state, we performed RNA sequencing of the *NF2^−/−^* hiPSCs. There were no significant differences in the expression levels of genes maintaining the pluripotent state between *NF2^−/−^* 1 (*n* = 3) and WT hiPSCs (*n* = 3) (Figure S2A, Supporting Information). These results suggest that mechanisms of endoderm specification, and not maintenance of the pluripotent state, were impaired in *NF2* ^−/−^ hiPSCs.

**Figure 2:**
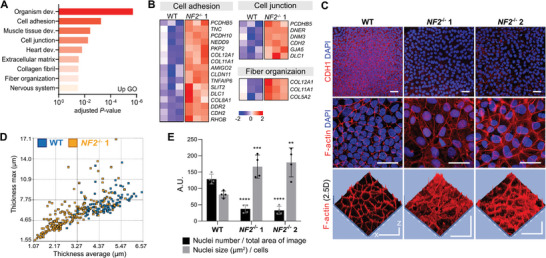
Loss of NF2 dysregulates cytoskeleton organization. A) Significantly up‐regulated gene ontology (GO) terms (adjusted *p *< 0.05) from the gene set enrichment analysis comparing *NF2^−/−^* 1 to WT hiPSCs. B) Heatmaps of differentially up‐regulated gene expression in hiPSCs, including genes related to cell adhesion (adjusted *p <* 0.001), cell junction (*p <* 0.006), and fiber organization (*p <* 0.03). Color denotes normalized gene expression values, with the darkest blue as the lowest expression and the darkest red as the highest. C) Immunostaining of WT and *NF2* ^−/−^ hiPSCs for cell membrane protein E‐cadherin (CDH1) and cytoskeletal F‐actin. Scale bars, 20 µm. Z‐axis in 2.5D, 200 nm. D) Comparison of WT and *NF2^−/−^* 1 maximum and average cell thickness using the HoloMonitor. Each dot represents individual cells. E) Comparison of the number of cells per µm^2^ and the area of nuclei per cell. A.U., arbitrary unit. ^**^ *p *< 0.01, ^***^ *p *< 0.001, ^****^ *p *< 0.0001. See “Quantification and Statistical Analysis” in the Experimental Section for statistics and experimental information.

Considering these findings, we analyzed all differentially expressed genes in the hiPSC RNA sequencing data to identify other potential effectors of endoderm specification failure in the *NF2^−/−^* 1 hiPSCs (Figure S2B, Supporting Information). We identified 254 differentially expressed genes (138 up‐ and 116 down‐regulated, |log_2_ fold‐change| >1, adjusted *p *< 0.05) in *NF2^−/−^* 1 compared to WT hiPSCs (Table S2A, Supplementary Table), then conducted a gene set enrichment analysis to determine how these transcriptomic differences were related to functional differences. Gene ontology (GO) analysis of up‐regulated gene sets highlighted organism development, mesoderm formation (muscle tissue and heart development), and cell‐cell interaction (cell adhesion and junction) (**Figure** **2**A). We further identified 18 dysregulated genes associated with embryo development (GO:0009790), including 14 up‐regulated and 4 down‐regulated genes, in *NF2* ^−/−^ 1 hiPSCs (Figure S2C, Supporting Information). Among these, *COL8A1, COL11A1* and *COL12A1*, which are critical for endoderm formation (GO:0001706), were dysregulated in *NF2* ^−/−^ 1 hiPSCs (Figure S2D, Supporting Information).

*DDR2*, a cell surface receptor for fibrillar collagen,^[^ ^15^ ^]^ identified as one of the most significantly up‐regulated genes (Figure 2B, Table S2A, Supplementary Table), functions in cell differentiation and regulation of the extracellular matrix. The *NF2* gene product merlin acts as a membrane cytoskeleton‐linking protein essential for regulating cell‐cell contact‐dependent proliferation inhibition.^[^ ^16^ ^]^ Notably, *DDR2* knock‐down in a human neuroblastoma cell line resulted in significant induction of *NF2*,^[^ ^17^ ^]^ although the precise mechanism remains unclear.

Cytomechanical properties such as cell shape, size, elasticity, and adhesion are crucial in regulating stemness in pluripotent cells and are tightly connected to actin cytoskeletal dynamics.^[^ ^18^ ^]^ In normal cells, merlin is recruited to form a complex with E‐cadherin (CDH1), maintaining stable cell‐cell contacts and actin networks.^[^ ^16^ ^]^ Conversely, *NF2^−/−^* hiPSCs exhibited disrupted filamentous actin (F‐actin) arrangement and lacked stable CDH1 adherens junctions (Figure 2C; Figure S2E, Supporting Information). Interestingly, the loss of *NF2* affects not only the local distribution of CDH1 protein but also its abundance in cells (Figure S2F, Supporting Information). To explore the mechanisms underlying this reduction, we investigated factors potentially influencing *CDH1* expression in *NF2* ^−/−^ hiPSCs. RT‐qPCR results confirmed the significant upregulation of *COL8A1* (WT: 1.0 ± 0.34, *NF2* ^−/−^ 1: 2.1 ± 0.45, *NF2* ^−/−^ 2: 2.2 ± 0.37) and *COL12A1* (WT: 1.0 ± 0.031, *NF2* ^−/−^ 1: 1.5 ± 0.088, *NF2* ^−/−^ 2: 1.7 ± 0.070) in *NF2* ^−/−^ hiPSCs, alongside a reduction in *CDH1* expression (WT: 1.0 ± 0.19, *NF2* ^−/−^ 1: 0.28 ± 0.020, *NF2* ^−/−^ 2: 0.22 ± 0.11) (Figure S2G, Supporting Information). This finding is consistent with Menke et al.^[^ ^19^ ^]^ which reported that some collagens (i.e., collagen type I and III) in culture substrates reduce *CDH1* expression in pancreatic carcinoma cell lines. Additionally, the epithelial‐mesenchymal transition‐regulating transcription factors *SNAI2* (WT: 1.0 ± 0.28, *NF2* ^−/−^ 1: 4.1 ± 0.84, *NF2* ^−/−^ 2: 7.6 ± 2.5) and *TWIST1* (WT: 1.0 ± 0.17, *NF2* ^−/−^ 1: 2.5 ± 0.41, *NF2* ^−/−^ 2: 1.7 ± 0.13), which are well‐established repressors of *CDH1* expression,^[^ ^20^ ^]^ were up‐regulated in *NF2* ^−/−^ hiPSCs (Figure S2G, Supporting Information). Therefore, *NF2*‐mediated regulation of *CDH1* may be via the promotion of *CDH1* suppressor genes.

Furthermore, *NF2^−/−^* hiPSCs developed cytoskeletal tension stress fibers, which correlated with increased expression of extracellular matrix genes^[^ ^21^ ^]^ (Table S2A, Supplementary Table). Additionally, the mean (*t*‐test, *p <* 0.0001) and maximum thickness (*p <* 0.0001) of individual cells were significantly thinner in *NF2* ^−/−^ 1 hiPSCs (2.8 ± 1.2 µm and 5.3 ± 2.7 µm, respectively) compared to WT hiPSCs (4.5 ± 0.8 µm and 7.1 ± 1.5 µm) (Figure 2D). Lastly, we observed that *NF2^−/−^* hiPSCs had significantly fewer nuclei per unit area (WT: 128.5 ± 15.2, *NF2^−/−^* 1: 37.3 ± 12.5, *NF2^−/−^* 2: 57.25 ± 6.1 per 18 211.5 µm^2^) and larger average nucleus size per cell (81.5 ± 10.6, 166.8 ± 35.4, and 141.3 ± 19.4 µm^2^, respectively) compared to WT hiPSCs (Figure 2E). Thus, *NF2* deletion leads to alterations in cytomechanical properties such as flattered cell shape, increased surface adherence, and fewer but larger nuclei.

### 2.3 Loss of *NF2* Promotes YAP1 Nuclear Localization and Hinders Endoderm Differentiation

**Figure 3:**
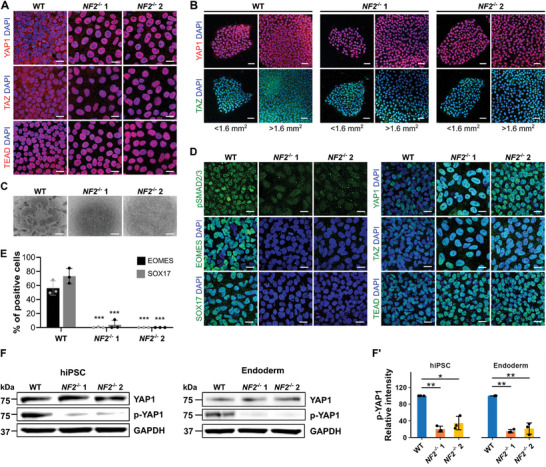
Loss of *NF2* results in YAP1 nuclear translocation and prevents endoderm differentiation. A) Immunostaining for downstream effectors of the Hippo pathway YAP1, TAZ, and TEAD in hiPSCs. Scale bars, 20 µm. B) Immunostaining for YAP1 and TAZ in hiPSC colonies of sizes < 1.6 mm^2^ and > 1.6 mm^2^. Scale bars, 50 µm. C) Representative phase contrast images of endoderm lineage cells generated from WT, *NF2* ^−/−^ 1, and *NF2* ^−/−^ 2 hiPSCs. Scale bars, 500 µm. D) Immunocytochemistry of endoderm and Hippo effector proteins in WT, *NF2* ^−/−^ 1, and *NF2* ^−/−^ 2 cells after endoderm differentiation. Scale bars, 20 µm. E) Proportions of cells positive for EOMES and SOX17 protein expression shown in Figure 3D. Y axis represents % of positive cells among all cells indicated by DAPI. F) Western blot showing relatively similar expression levels of YAP1 across hiPSC and endoderm‐differentiated cell types. In contrast, Ser127 phosphorylated (p)‐YAP1 (Ser127) was reduced in *NF2* ^−/−^ compared to WT cells. Protein sizes: GAPDH (37 kDa), p‐YAP1 (75 kDa), YAP1 (75 kDa). F’) Quantification of p‐YAP1 Western blot in WT and *NF2* ^−/−^ hiPSCs and after endoderm differentiation. ^*^ *p *< 0.05, ^**^ *p *< 0.01, ^***^ *p *< 0.001. See “Quantification and Statistical Analysis” in the Experimental Section for statistics and experimental information.

Disorganized actin fibers promote YAP1/TAZ nuclear enrichment independent of the core Hippo kinases,^[^ ^22^ ^]^ and, in meningioma cells, the knockdown of *NF2* RNA increases YAP1 expression and nuclear localization via the Hippo pathway.^[^ ^23^ ^]^ We similarly observed that YAP1 and TAZ were mainly located in the nuclei of *NF2* ^−/−^ 1 and *NF2* ^−/−^ 2, but not WT, hiPSCs (**Figure** **3**A). These findings were consistent regardless of colony size (Figure 3B), as the location of YAP1 could be affected by colony size and cell density.^[^ ^24^ ^]^ In contrast, the WT hiPSCs displayed both cytoplasmic and nuclear expression of YAP1/TAZ, although the localization pattern varied. Unlike YAP1 and TAZ, the co‐factor TEAD was localized in the nucleus across cell types.

Given that YAP1/TAZ are central hubs controlling gene expression during embryogenesis,^[^ ^25^ ^]^ we hypothesized that their nuclear translocation in *NF2^−/−^* hiPSCs might be involved in the failure to form endoderm. To test this, we compared WT and *NF2^−/−^* cells under endoderm lineage differentiation conditions in vitro. Unlike WT cells, which developed the expected endoderm‐specific aggregated colony formation, *NF2^−/−^* 1 and *NF2^−/−^* 2 cells displayed completely flattened morphology, lacking these characteristic structures (Figure 3C). Furthermore, the endoderm differentiated WT cells were positive for markers of the onset of endoderm specification and differentiation^[^ ^26^ ^]^ such as EOMES (56.20% ± 10.11% of cells) and SOX17 (73.08% ± 10.81%) (Figure 3D,E; Figure S3A,B, Supporting Information). In contrast, very few *NF2^−/−^* cells were positive for EOMES (*NF2^−/−^* 1: 0.38% ± 0.63%, *NF2^−/−^* 2: 0.04% ± 0.05%) or SOX17 (*NF2^−/−^* 1: 3.88% ± 6.34%, *NF2^−/−^* 2: 0.00% ± 0.00%) after differentiation. Similar to the hiPSC stage, YAP1/TAZ were still exclusively located in the nuclei of *NF2^−/−^* cells under endoderm differentiation conditions.

Notably, while total YAP1 protein levels were similar between WT and *NF2^−/−^* cells, phosphorylated YAP1 at Ser127 (p‐YAP1)—a modification that promotes cytoplasmic retention—was significantly reduced in both *NF2^−/−^* clones in their hiPSCs state (*NF2^−/−^* 1: 20.13% ± 7.10%, *NF2^−/−^* 2: 15.24% ± 4.13% compared to WT) and after endoderm differentiation (*NF2^−/−^* 1: 34.69% ± 16.05%, *NF2^−/−^* 2: 21.52% ± 13.49% compared to WT) (Figure 3F,F’). Taken together, these results suggest that loss of *NF2* facilitates the nuclear accumulation of YAP1 by attenuating YAP1 Ser127 phosphorylation and may hinder endoderm formation.

### 2.4 *YAP1* Knockdown Rescues Endoderm Differentiation

**Figure 4:**
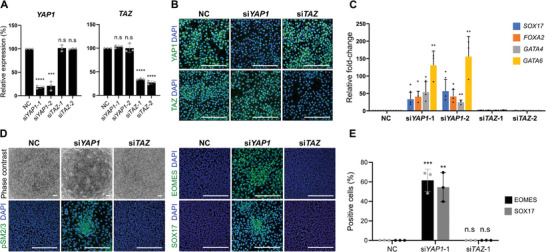
Rescue of endoderm differentiation by *YAP1* knockdown. A) Relative expression of *YAP1* and *TAZ* mRNA in *NF2^−/−^* 1 cells on day 1 of endoderm differentiation after treatment with si*YAP1* or si*TAZ* day 0–1. The average expression in negative control siRNA‐treated (NC) cells was set to 100. B) Representative immunostaining images showing YAP1 and TAZ protein expression on day 1 of endoderm differentiation after treatment with si*YAP1* or si*TAZ* day 0–1. C) siRNA‐mediated knockdown of *YAP1* (si*YAP1*‐1 and si*YAP1*‐2) but not *TAZ* (si*TAZ*‐1 and si*TAZ*‐2) restores expression of endoderm‐related genes in *NF2^−/−^* 1 cells under endoderm differentiation conditions on day 4. Application of *YAP1* and *TAZ* siRNA was between day 0 and 1. D) Knockdown of *YAP1*, but not *TAZ*, restores endoderm morphology and pSMAD2/3, EOMES, and SOX17 protein expression in *NF2^−/−^* 1 cells on day 4 of endoderm differentiation. Application of *YAP1*‐1 and *TAZ*‐1 siRNA was between day 0 and 1. Scale bars, 200 µm. E) Proportions of cells positive for EOMES and SOX17 protein shown in Figure 4D. Y axis represents % of positive cells among all cells indicated by DAPI. n.s.: *p* > 0.05, ^*^ *p* < 0.05, ^**^ *p* < 0.01, ^***^ *p *< 0.001, ^****^ *p *< 0.0001. See “Quantification and Statistical Analysis” in the Experimental Section for statistics and experimental information.

To investigate whether suppression of excessive nuclear YAP1 or TAZ can restore the endoderm differentiation capability in *NF2^−/−^* cells, we separately knocked down *YAP1* or *TAZ* using 4 different small interfering RNA (siRNA) oligos (i.e., 2 for each gene). Compared to negative control (NC) siRNA transfection, si*YAP1*‐1 and si*YAP1*‐2 oligos into *NF2^−/−^* 1 cells reduced *YAP1* mRNA by 18.3% ± 3.1% and 20.9% ± 9.6%, respectively, while si*TAZ*‐1 and si*TAZ*‐2 oligos reduced *TAZ* mRNA by 34.6% ± 1.4% and 27.5% ± 3.5% (**Figure** **4**A; Figure S4A,B, Supporting Information). These mRNA knockdown effects were reproducible using *NF2^−/−^* 2 cells for both *YAP1* (si*YAP1*‐1: 10.7% ± 5.0%, si*YAP1*‐2: 11.5% ± 7.4%) and *TAZ* (si*TAZ*‐1: 27.6% ± 4.3%, si*YAP1*‐2: 22.4% ± 7.5%) (Figure S4C, Supporting Information). Additionally, we observed that protein levels of YAP1 (NC: 92.8% ± 0.59%, si*YAP1*‐1: 47.1% ± 2.0%, si*TAZ*‐1: 95.0% ± 1.3%) and TAZ (NC: 96.8% ± 0.69%, si*YAP1*‐1: 95.1% ± 0.6%, si*TAZ*‐1: 48.1% ± 6.4%) were reduced following application of si*YAP1*‐1 and si*TAZ*‐1, respectively (Figure 4B).

Interestingly, the knockdown of *YAP1*, but not *TAZ*, rescued mRNA expression of endoderm marker genes in *NF2^−/−^* cells under endoderm differentiation conditions (Figure 4C; Figure S4D, Supporting Information). Endoderm‐like morphology and EOMES (NC: 0.09% ± 0.15%, si*YAP1*‐1: 61.6% ± 11.5%, si*TAZ*‐1: 0.02% ± 0.03%) and SOX17 (NC: 0.02% ± 0.03%, si*YAP1*‐1: 54.5% ± 14.9%, si*TAZ*‐1: 0.07% ± 0.13%) proteins also reappeared in the *NF2^−/−^* cells following application of *YAP1* siRNA (Figure 4D,E). We then examined whether endoderm formation in *NF2^−/−^* cells could be recovered by decoupling the YAP1‐TEAD complex with 62.5 or 125 nM verteporfin, a benzoporphyrin derivative.^[^ ^27^ ^]^ There were no significant changes in the expression of endoderm markers at either verteporfin concentration compared to the control, and higher concentrations induced cytotoxicity (Figure S4E,F, Supporting Information). In summary, the knockdown of *YAP1* alone is sufficient to restore the capacity for endoderm formation in *NF2^−/−^* cells and YAP1 acts independently of the co‐activators TAZ and TEAD to impair endoderm lineage specification.

### 2.5 *NF2* Deletion Leads to a Myofibroblast‐Like Fate Instead of Endoderm Formation Via the TGFβ Pathway

**Figure 5:**
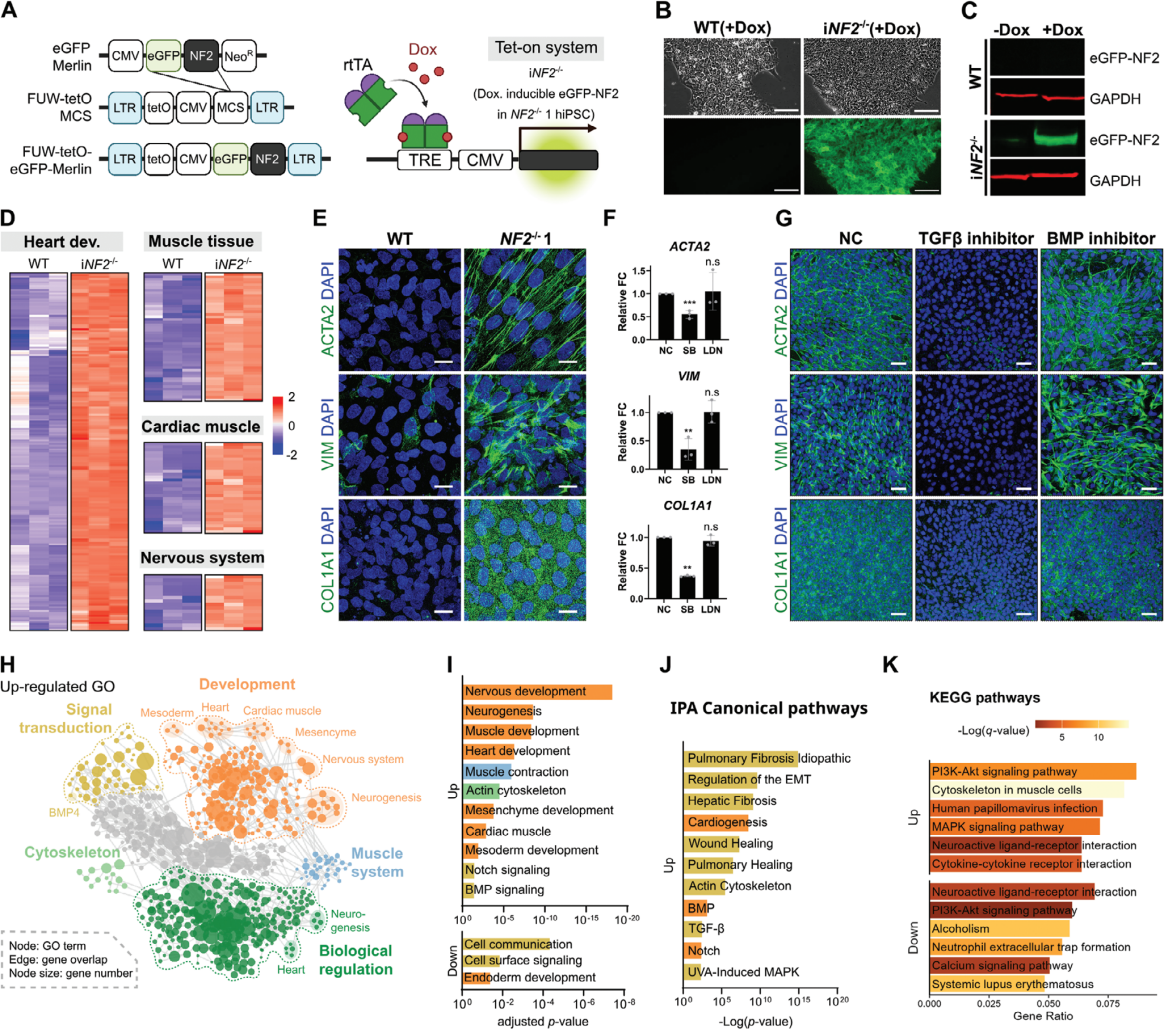
Loss of *NF2* leads differentiating cells to a myofibroblast‐like fate through the TGFβ pathway. A) Schematic of the generation of an FUW‐tetO‐eGFP‐NF2 construct for establishing doxycycline (Dox)‐inducible eGFP‐NF2‐expressing hiPSC lines (i*NF2* ^−/−^). The reverse tetracycline transactivator (rtTA) protein is capable of binding the Tetracycline Response Element (TRE; several TetO sequences with a minimal promoter) only in the presence of Dox. The introduction of Dox to the Tet‐on system initiates the transcription of eGFP‐NF2. B) Representative phase contrast and fluorescence live cell images of WT and i*NF2* ^−/−^ hiPSCs in the presence of Dox (0.5 µg mL^−1^). Scale bars, 100 µm. C) Western blot images of eGFP‐NF2 and GAPDH protein expression with or without Dox in WT and i*NF2* ^−/−^ hiPSCs. D) Heatmaps of gene expression in i*NF2* ^−/−^ (‐Dox) and WT cells after endoderm differentiation, including those related to heart development (adjusted *p *= 2.96E‐07), muscle tissue development (1.10E‐06), cardiac muscle tissue development (8.34E‐04), and nervous system development (6.22E‐18). Color denotes normalized gene expression values, with the darkest blue as the lowest expression and the darkest red as the highest. E) WT and *NF2^−/−^* 1 cells immunostained with myofibroblast markers (ACTA2, VIM, and COL1A1) under endoderm differentiation conditions. Scale bars, 20 µm. F) Reduction of mRNA expression of myofibroblast‐related genes following application of TGFβ inhibitor SB431542 (SB), but not BMP inhibitor LDN193189 (LDN), compared to negative control (NC; DMSO‐treated) under endoderm differentiation conditions. G) Reduction in the expression of myofibroblast marker proteins in *NF2^−/−^* 1 cell after treatment with TGFβ inhibitor SB, but not BMP inhibitor LDN, under endoderm differentiation conditions. Scale bars, 50 µm. H) Gene set enrichment analysis of i*NF2^−/−^* (‐Dox) compared to WT mRNA at the endoderm stage, visualized by network using Cytoscape. I) Gene ontology analysis of the endoderm cells, illustrating significantly up‐ and down‐regulated terms in i*NF2^−/−^* (‐Dox) compared to WT cells. J) Ingenuity Pathway Analysis (IPA) showing up‐regulated canonical pathways in i*NF2^−/−^* (‐Dox) compared to WT cell mRNA after endoderm differentiation. K) Kyoto Encyclopedia Genes and Genomes (KEGG) pathway analysis of i*NF2^−/−^* (‐Dox) cells following endoderm differentiation. n.s.: *p* > 0.05, ^*^ *p*  <  0.05, ^**^ *p* < 0.01, ^***^ *p* < 0.001. See “Quantification and Statistical Analysis” in the Experimental Section for statistics and experimental information.

Next, we developed a doxycycline (Dox)‐inducible *NF2* expression system tagged with eGFP in the *NF2^−/−^* 1 hiPSC line (**Figure** **5**A; Figure S5A, Supporting Information), hereafter referred to as i*NF2^−/−^*, to investigate the transcriptomic effects of both *NF2* loss‐of‐function and gain‐of‐function after endoderm differentiation. In the presence of Dox, *NF2* mRNA induction was observed in a dose‐dependent manner (Figure S5B, Supporting Information). A Dox concentration of 0.5 µg mL^−1^ was selected for subsequent experiments, as it ensured sufficient eGFP‐NF2 expression without inducing cytotoxicity. eGFP‐NF2 protein expression in the hiPSC state was confirmed by live cell fluorescence imaging (Figure 5B) and by Western blot (Figure 5C). As expected, eGFP‐NF2 was mainly localized at the leading edge or at the cell‐cell adhesion points.

Then, we performed RNA sequencing analysis on the i*NF2^−/−^* cells differentiated into endoderm in the presence (+Dox) or absence (‐Dox) of Dox, as well as on WT endoderm cells. The amount of *NF2* mRNA was significantly higher in i*NF2^−/−^* (+Dox) than in i*NF2^−/−^* (‐Dox) and was within a statistically similar range to that of the isogenic control WT (Figure S5C, Supporting Information). In the i*NF2^−/−^* (‐Dox) cells, there were 9265 significantly differentially expressed genes (DEGs) compared to WT after endoderm differentiation, with 2048 genes up‐regulated and 7217 genes down‐regulated. Subsequently, we asked how *NF2* deletion impacts the ultimate cell fate decision and resulting cell identities. Genes related to the development of the heart, muscle tissue, cardiac muscle tissue, and nervous system were up‐regulated in the i*NF2^−/−^* (‐Dox) cells (Figure 5D). This finding is consistent with previous reports that blocking endogenous SMAD2/3 activation redirects the primitive streak toward mesodermal and neural crest fates.^[^ ^28^ ^]^ Next, we assessed genes associated with mesodermal and ectodermal differentiation, focusing on those associated with YAP1 or TAZ. Six candidate genes were selected from the top 30 DEGs (Table S2B, Supplementary Table), and their expressions were cross‐validated in *NF2* ^−/−^ 1 cells following *YAP1* or *TAZ* knockdown during endoderm differentiation. *FOXC2* (GO terms: Muscle structure development, Heart development, Cardiac muscle) was down‐regulated by only si*YAP1* and other genes such as *BMP4* (Nervous system, Heart development), *FEZF2* (Nervous system), *XIRP1* (Muscle structure development, Heart development, Cardiac muscle), and *LHX5* (Nervous system) were down‐regulated by only si*TAZ*. However, *NKX2‐5* (Muscle structure development, Heart development, Cardiac muscle) was regulated by both *YAP1* and *TAZ* (Figure S5D, Supporting Information). These results demonstrate that *YAP1* or *TAZ*, either independently or together, may influence cell fate toward these lineages in *NF2^−/−^* cells.

In contrast with the transcriptomic data suggesting *NF2^−/−^* cells up‐regulated these genes, there was less evidence at the protein level. For example, semi‐quantitative analysis of immunostained images showed that only 4.29% ± 4.46% of the *NF2^−/−^* 1 cells and 0.539% ± 0.543% of the *NF2^−/−^* 2 cells were positive for NKX2‐5, while LHX5 expression was too weak to quantify (Figure S5E, Supporting Information). However, the majority of *NF2^−/−^* 1 and *NF2^−/−^* 2 cells expressed both alpha‐smooth muscle actin (ACTA2, 100% ± 0%, 100% ± 0%, respectively), vimentin (VIM, 99.3% ± 0.298%, 97.3% ± 1.33%), and collagen type 1 (COL1A1 [a key component of the extracellular matrix], 91.4% ± 3.62%, 90.7% ± 0.439%) compared to WT (ACTA2: 2.76% ± 0.760%, VIM: 9.74% ± 3.61%, COL1A1: 2.38% ± 0.979%) (Figure 5E; Figure S5E, Supporting Information). In addition, *NF2^−/−^* 1 and *NF2^−/−^* 2 cells did not express smooth muscle cell marker desmin (DES) or vascular endothelial marker CD31 (Figure S5E, Supporting Information). As the cells also had a spindle‐shaped morphology with long, thin cytoplasmic extensions, we surmised they most closely resembled myofibroblasts.^[^ ^29^ ^]^

Activated myofibroblasts are the primary producers and organizers of extracellular matrix proteins during wound healing (i.e., fibrogenesis),^[^ ^30^ ^]^ and disruption of their tightly controlled role can result in pathological scarring of organs or tumor stroma remodeling.^[^ ^31^ ^]^ TGFβ^[^ ^32^ ^]^ and BMP^[^ ^33^ ^]^ ligands or their downstream effectors are involved in fibrosis, and *TGFB2* and *BMP4* mRNA were highly expressed in *NF2^−/−^* 1 cells (Table S2B, Supplementary Table). Therefore, we investigated the effect of inhibitors of TGFβ (SB431542 [SB]) and BMP (LDN193189 [LDN]) on the expression of fibrogenesis‐related genes and proteins in *NF2^−/−^* 1 cells. We observed that, compared to NC, the mRNA levels of myofibroblast‐related genes, such as *ACTA2*, *COL1A1*, and *VIM*, were decreased in *NF2* ^−/−^ 1 cell following the application of the TGFβ inhibitor, but not the BMP inhibitor. Specifically, relative expression levels were as follows: *ACTA2* (NC: 1.0 ± 0, SB: 0.55 ± 0.087, LDN: 1.1 ± 0.41), *VIM* (NC: 1.0 ± 0, SB: 0.35 ± 0.19, LDN: 1.0 ± 0.20), and *COL1A1* (NC: 1.0 ± 0, SB: 0.37 ± 0.012, LDN: 0.94 ± 0.086) (Figure 5F). Similarly, reductions in myofibroblast‐related proteins ACTA2 (NC: 99.7% ± 0.258%, SB: 15.5% ± 2.25%, LDN: 98.6% ± 0.933%), VIM (NC: 98.1% ± 0.181%, SB: 10.1% ± 1.45%, LDN: 97.5% ± 0.459%), COL1A1 (NC: 92.6% ± 1.41%, SB: 22.2% ± 5.64%, LDN: 90.7% ± 1.40%) were observed under SB‐treated conditions (Figure 5G).

The genetic differences between WT and i*NF2^−/−^* (‐Dox) cells after endoderm differentiation were further illustrated by a gene set enrichment analysis (Figure 5H). The GO terms from the down‐regulated gene set included “Cell communication” and “Cell surface signaling pathway” (Figure 5I), supporting that impaired cell‐cell interaction dynamics contribute to the failure of endoderm differentiation due to *NF2* deletion. Ingenuity Pathway Analysis (IPA) revealed activation of canonical pathways associated with fibrosis and wound healing (Figure 5J). Kyoto Encyclopedia of Genes and Genomes (KEGG) pathway analysis highlighted an up‐regulation of cytoskeleton‐related gene expression in muscle cells (Figure 5K). Taken together, these findings indicate that, in the absence of *NF2*, the TGFβ pathway redirects *NF2^−/−^* hiPSCs toward a myofibroblast‐like fate even under endoderm differentiation conditions.

### 2.6 Endoderm Formation is Restored by Forced Ectopic *NF2* Expression in *NF2^−/−^* Cells

**Figure 6:**
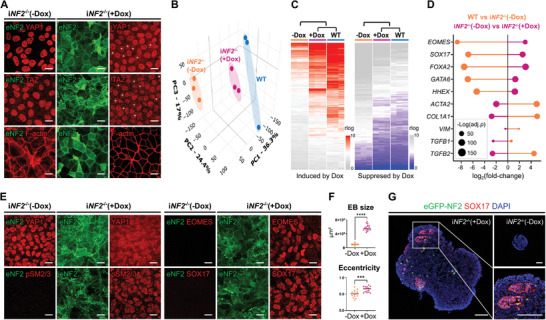
Rescue of endoderm differentiation by inducible expression of *NF2* in *NF2^−/−^* cells. A) Immunostaining for YAP1, TAZ, and F‐actin in i*NF2* ^−/−^ hiPSCs with Dox (0.5 µg mL^−1^) and without Dox. Scale bars, 20 µm. B) 3D principal component analysis plot of WT versus i*NF2* ^−/−^ (‐Dox) versus i*NF2* ^−/−^ (+Dox) after endoderm differentiation from RNA sequencing data. C) Heatmaps showing the top 200 significantly induced and suppressed genes in i*NF2* ^−/−^ (‐Dox) versus i*NF2* ^−/−^ (+Dox) versus WT cells after endoderm differentiation and their hierarchical clustering. D) Expression of genes related to endoderm and myofibroblasts in WT, i*NF2* ^−/−^ (‐Dox), and i*NF2* ^−/−^ (+Dox) cells after endoderm differentiation. The size of dots represents ‐Log(adjusted *p*‐value). E) Immunostaining of i*NF2* ^−/−^ (‐Dox) and i*NF2* ^−/−^ (+Dox) cells to YAP1, pSMAD2/3, and endoderm markers under endoderm differentiation. Scale bars, 20 µm. F) Measurement of embryoid body (EB) size (*n* = 20) and eccentricity (*n* = 15). EBs with greater eccentricity have less curvature (i.e., a perfect circle has an eccentricity of zero). G) Immunostaining of EBs with and without Dox using endoderm marker SOX17. Scale bars, 100 µm. ^***^ *p *< 0.001, ^****^ *p* < 0.0001. See “Quantification and Statistical Analysis” in the Experimental Section for statistics and experimental information.

We then investigated whether ectopic *NF2* induction could rescue endoderm formation and other characteristics related to *NF2* deficiency in the knockout cells. Indeed, semi‐quantitative analysis of immunostaining images showed that 31.0% ± 7.27% i*NF2^−/−^* (+Dox) hiPSCs had diminished nuclear YAP1 expression compared to only 1.45% ± 1.27% i*NF2^−/−^* (‐Dox) hiPSCs. Similarly, 25.6% ± 4.59% i*NF2^−/−^* (+Dox) hiPSCs showed decreased nuclear TAZ expression compared to 3.02% ± 1.51% i*NF2^−/−^* (‐Dox) hiPSCs. Additionally, the F‐actin arrangement appeared more organized in i*NF2^−/−^* (+Dox) (**Figure** **6**A). A 3D principal component analysis plot demonstrated that forced *NF2* expression prominently shifted the transcriptome pattern of the i*NF2^−/−^* (+Dox) cells toward that of WT (Figure 6B). After endoderm differentiation, a total of 6221 DEGs were identified in i*NF2^−/−^* (+Dox) cells, with 1125 genes up‐regulated and 5096 genes down‐regulated compared to i*NF2^−/−^* (‐Dox) cells. We then selected the top 200 DEGs that were either induced or suppressed after *NF2* induction. Hierarchical clustering analysis depicted that the expression levels of these genes in i*NF2^−/−^* (+Dox) returned to the normal range, similar to WT cells (Figure 6C). Specifically, endoderm‐related genes (*EOMES*, *SOX17*, *FOXA2*, *GATA6*, and *HHEX*), which were decreased in i*NF2^−/−^* (‐Dox) cells, rebounded after *NF2* induction. Conversely, the expression of genes related to fibrosis (*ACTA2*, *COL1A1, and VIM*) and possible ligands that activate the TGFβ signaling pathway (*TGFB1*, *TGFB2*), were suppressed following *NF2* induction (Figure 6D).

Immunostaining was performed to confirm the restoration of normal protein production and localization induced by forced *NF2* expression at the endoderm formation stage. i*NF2^−/−^* (+Dox) cells exhibited lower nuclear expression of YAP1 compared to i*NF2^−/−^* (‐Dox) cells (Figure 6E). Specifically, 53.0% ± 4.4% of i*NF2^−/−^* (+Dox) cells and 95.6% ± 0.49% of i*NF2^−/−^* (‐Dox) cells were positive for YAP1 only in the nucleus. In addition, the expression of key endoderm markers EOMES (77.9% ± 12.8%) and SOX17 (82.3% ± 7.4%) was recovered in i*NF2^−/−^* (+Dox) endoderm differentiated cells (Figure 6E).

To further test the restoration of endoderm lineage development, we created 3D embryoid bodies (EBs) to mimic and recapitulate aspects of blastocyst formation and gastrulation.^[^ ^34^ ^]^ Morphologically, i*NF2^−/−^* (+Dox) EBs displayed heterogenous multi‐layered cell‐cell interactions, while i*NF2^−/−^* (‐Dox) EBs remained compacted cell aggregations (Figure S6A, Supporting Information).^[^ ^35^ ^]^ Also, the average size of i*NF2^−/−^* (+Dox) EBs (0.572 ± 0.083 mm^2^) was significantly larger than that of i*NF2^−/−^* (‐Dox) EBs, (0.098 ± 0.022 mm^2^) (Figure 6F). During *Xenopus laevis* early embryo development, NF2 plays an important role in axial pattern formation.^[^ ^36^ ^]^ In this context, the mean eccentricity of EBs was significantly higher in i*NF2^−/−^* (+Dox, 0.64 ± 0.08) compared to i*NF2^−/−^* (‐Dox, 0.51 ± 0.11). Notably, all i*NF2^−/−^* (+Dox) EBs included SOX17 (21/21 EBs)‐ and EOMES (9/9 EBs)‐positive endoderm lineage cells. In contrast, only one of 10 i*NF2^−/−^* (‐Dox) EBs had weak SOX17 expression and none were positive for EOMES (0/11 EBs) (Figure 6G; Figure S6B, Supporting Information). We further confirmed that *NF2* deletion did not affect ectoderm or mesoderm differentiation, as demonstrated by immunostaining of NESTIN, PAX6 (ectoderm), TBXT, and ACTA2 (mesoderm) (Figure S6C, Supporting Information).

## 3 Discussion

In contrast to the well‐known function of *NF2* as a tumor suppressor gene,^[^ ^37^ ^]^ its specific role in human development has long been elusive. In this study, we identified *NF2* as essential for human endoderm specification and formation by using *NF2* knockout hiPSCs cultured via monolayer direct differentiation and 3D self‐organized EB methods. We found that loss of *NF2* abolished the capacity of hiPSCs to generate endoderm lineage cells, which was attributed to the nuclear translocation of YAP1 and subsequent inactivation of the Activin/Nodal pathway required for expression of the endoderm genes *SOX17*, *FOXA2*, *GATA4*, and *GATA6*. Instead, hiPSCs lacking *NF2* were directed to a myofibroblast‐like fate under endoderm differentiation conditions. This was triggered by the activation of the TGFβ signaling pathway and expression of *ACTA2*, *VIM*, and *COL1A1*. Importantly, the *NF2* ^−/−^ cells’ capacity to form endoderm could be restored via the siRNA‐mediated knockdown of *YAP1* or the forced expression of *NF2* with a tet‐on system, placing *YAP1* repression under the control of *NF2* during this stage of human development.

Multiple studies are in line with our findings that the loss of *NF2* would disrupt the Hippo pathway during human endoderm formation, leading to aberrant gene expression due to excessive nuclear expression of YAP1. In mice, homozygous *Yap1* deletion leads to embryonic lethality shortly after gastrulation onset and Yap1 has lower expression within the region of the primitive streak destined to become endoderm,^[^ ^7d^ ^]^ highlighting its role in anterior‐posterior axis formation and importance in the formation of the 3 germ layers. Further, in vitro studies using human pluripotent stem cells exposed to activin demonstrated that loss of YAP1 enables differentiation toward anterior primitive streak and endoderm cell fate.^[^ ^38^ ^]^ There have also been a few prior attempts to generate *NF2‐*deficient hiPSCs for the purpose of studying tumorigenesis,^[^ ^39^ ^]^ improving diagnosis of neurofibromatosis type 2,^[^ ^40^ ^]^ or modeling *NF2*‐related schwannomas.^[^ ^41^ ^]^ A preprint by Catasús et al. reported that *NF2* ^+/−^ hiPSC lines demonstrated a normal capacity to differentiate into the 3 germ layers, but *NF2* ^−/−^ hiPSC failed to form EBs under standard protocol conditions.^[^ ^41^ ^]^ The altered differentiation capacity suggested *NF2* is involved in pluripotency, but the mechanisms were not identified. In our study, a detailed interrogation of the defect revealed it was due to a lack of expression of endoderm‐specifying genes resulting from YAP1 nuclear translocation.

Although YAP1 and TAZ share common features as paralog transcriptional regulators, they have distinct transcriptional partners and control nonidentical transcriptional programs.^[^ ^25^ ^]^ Notably, we found that YAP1 functions independently of its co‐activators TAZ and TEAD in inhibiting the Activin/Nodal pathway and attenuating endoderm lineage formation; blocking TAZ or decoupling YAP1 from TEAD was not sufficient to restore endoderm formation. As YAP1 lacks a DNA‐binding domain, it requires interaction with other transcription factors such as p73, RUNX1/2, TBX5, SMAD, or PAX3.^[^ ^42^ ^]^ Future studies are needed to identify the co‐activator of YAP1 in this context. A potential candidate may be RUNX2, which had 3.9 times higher mRNA expression in *NF2* ^−/−^ 1 cells compared to WT cells following endoderm differentiation, and has been recently reported to associate with YAP1 to regulate cardiac fibroblast proliferation.^[^ ^43^ ^]^

The Activin/Nodal pathway has previously been implicated in maintaining the pluripotent state and inducing the primitive streak during gastrulation, as well as specifying the anterior primitive streak.^[^ ^44^ ^]^ Additionally, inhibiting Activin/Nodal signaling in human embryonic stem cells has been reported to lead to loss of *NANOG* expression and promotion of neuroectodermal differentiation.^[^ ^45^ ^]^ However, we did not observe differential expression of pluripotent state‐related genes in the *NF2* ^−/−^ cells in this study, including *NANOG*, suggesting that this role of Activin/Nodal signaling was not affected at the hiPSC stage by the loss of *NF2*. YAP1/TAZ is also reported to be important for maintaining a pluripotent state^[^ ^46^ ^]^ (e.g., YAP1/TAZ can form transcriptional complexes with SMAD2/3 and OCT4 to regulate pluripotent stage‐associated genes such as *NANOG* [46b]). Thus, the nuclear translocation of YAP1/TAZ in *NF2* ^−/−^ cells may contribute to the preservation of an undifferentiated state. Furthermore, the Activin/Nodal pathway is not the sole determinant of primitive streak induction and mesendoderm specification. For example, BMP4 alone can induce posterior primitive streak development during mouse embryonic stem differentiation,^[^ ^47^ ^]^ and inhibitors of Activin/Nodal signaling in the presence of BMP4 and FGF2 promote a mesoderm fate in human embryonic stem cells.^[^ ^48^ ^]^ Studies involving the loss of *Nodal* mRNA synthesis in mouse embryos have demonstrated that although there is no morphological evidence of complete primitive streak formation, these embryos still contain cells expressing posterior mesoderm markers.^[^ ^49^ ^]^ Consistent with previous findings, we observed the induction of mesendoderm cells expressing the pan‐primitive streak marker TBXT in *NF2* ^−/−^ EBs (Figure S6C, Supporting Information). Thus, the capacity for mesoderm induction by *NF2* ^−/−^ cells may be unaffected due to *BMP4* up‐regulation, although future studies are suggested to determine whether this extends to all mesodermal subtypes, particularly paraxial mesoderm as the most anterior part of the primitive streak.

In addition to elucidating the developmental importance of *NF2*, our study may provide insight into the potential mechanisms underlying pathological fibrosis in patients with inherited or sporadic *NF2* mutations. In alignment with *NF2’*s role as a tumor suppressor, somatic *NF2* mutations can prompt biallelic inactivation in tissues that promote neoplasia, and the risk is higher for patients who have already inherited one mutant *NF2* allele.^[^ ^37^ ^]^ The hallmark of *NF2‐*related schwannomatosis is bilateral vestibular schwannoma and these patients also have high rates of meningioma, leading to *NF2* being one of the first gene mutations implicated in meningioma formation.^[^ ^50^ ^]^ These tumors are commonly fibroblastic.^[^ ^51^ ^]^ Myofibroblasts, similar to those generated via TGFβ signaling in our *NF2* ^−/−^ cells, could contribute to fibrosis development in *NF2‐*related schwannomatosis and other tumors. Furthermore, prior studies have established the involvement of the Hippo pathway and YAP1 in multiple fibrotic diseases,^[^ ^52^ ^]^ myofibroblast differentiation,^[^ ^53^ ^]^ and cancer‐associated fibroblast generation.^[^ ^54^ ^]^ Understanding this mechanism is particularly important in the context of vestibular schwannoma, where fibrosis in the tumor microenvironment is associated with a greater risk of hearing loss.^[^ ^55^ ^]^ The present results provide a foundation for future investigations into the broader functions of *NF2* across different tissues and contexts, spanning human embryogenesis to disease processes in later life, and may provide valuable insight for future therapeutic strategies.

## 4 Experimental Section

### Generation of hiPSC Lines

The hiPSC lines *NF2^−/−^* 1, *NF2^−/−^* 2, *NF2^−/+^*, and i*NF2^−/−^* were generated in the laboratory, as described below, and the WT hiPSC were purchased from WiCell (WiCell Research Institute, UCSD112i‐2‐11). WT hiPSCs used for generation of *NF2^−/−^* 1, *NF2^−/−^* 2 and i*NF2^−/−^* were generated from *NF2^−/−^* 1. *NF2^−/+^* was generated from a patient with neurofibromatosis type 2 and details were described in the next section. The sequence information of guide RNA for *NF2* knockout hiPSC generation and genomic DNA PCR primers for knockout validation is available in Table S3 (Supplementary Table). The deletion of exon 2–4 region was confirmed by Sanger sequencing. The details are available from the Harvard Stem Cell Institute iPS Core Facility (https://ipscore.hsci.harvard.edu/genome‐editing‐services).

To rule out the possibility of a CRISPR/Cas9 off‐targeting effect during the generation of *NF2^−/−^* hiPSCs, the results of whole genome sequencing of wildtype and *NF2^−/−^* 1 hiPSCs were compared via somatic variant calling analysis (Figure S7A, Supporting Information). Variations including insertions and deletions (indels), single nucleotide polymorphisms, translocations, and transversions in coding or noncoding sequences between wildtype and *NF2^−/−^* 1 hiPSCs were investigated (Figure S7B,C, Supporting Information). The mutations found in pluripotency‐ or endoderm differentiation‐related genes in *NF2^−/−^* 1 showed minimal evidence of CRISPR/Cas9 off‐target (Table S4, Supplementary Table).

### hiPSCs from Patients with Neurofibromatosis Type 2 (*NF2^−/+^*)

Primary dermal fibroblasts were collected from a patient with neurofibromatosis type 2 at Massachusetts Eye and Ear (MEE) in Boston, MA, USA. Biopsied skin was collected into a transport medium of Dulbecco's Modified Eagle Medium (DMEM; Gibco) with 1% Penicillin‐Streptomycin‐Glutamine (PSG; Corning). Samples were washed twice in Phosphate‐Buffered Saline (PBS; Gibco) with 10% PSG, cut with a scalpel (Feather) into the smallest pieces possible, and plated onto a gridded p60 cell culture dish dermis side down. The plated tissue was incubated at 37 °C for 15 min to promote adherence to the dish. DMEM with 10% FBS (Sigma) and 1% PSG was gently added over the tissue pieces.

One day before transduction, 1.5 × 10^5^ mycoplasma‐free fibroblasts were seeded per well in a 6‐well plate coated with 0.1% gelatin and cultured in a fibroblast medium. On the day of transduction, cells were transduced with the CytoTune‐iPS 2.0 Sendai Reprogramming Kit (Invitrogen) in fibroblast medium at multiplicity of infection (MOI) of 3 for *Klf4* and MOI of 5 for *Klf4–Oct3/4–Sox2* and *cMyc*. The medium was replaced with fresh fibroblast medium daily. Five days post‐transduction, 2.5 × 10^5^ cells were transferred to 10 cm irradiated mouse embryonic fibroblasts culture dishes. After culturing in fibroblast media overnight, the medium was replaced daily with DMEM/F12 supplemented with 20% KnockOut Serum Replacement, 2 mM L‐Glutamine, 1x MEM Non‐Essential Amino Acids Solution, 55 µM 2‐Mercaptoethanol, and 10 ng mL^−1^ bFGF (all Gibco). The hiPSC colonies were picked for expansion and characterization from 18–25 days after transduction.

### Transgenic hiPSC Lines with Doxycycline‐Inducible *NF2* Gene Expression (i*NF2^−/−^*)

Genomic integration of a reverse tetracycline‐controlled transactivator (rtTA) cassette in *NF2^−/−^* 1 hiPSC line was achieved by a transposon/transposase system. A PiggyBac transposon (pPB) containing an rtTA‐expressing hygromycin‐selectable cassette under the control of the CAG promoter (pPB‐CAG‐rtTA‐IRES‐Hygro, Addgene, #102423) was co‐transfected with mPiggyBac transposase (mPB).^[^ ^56^ ^]^ Approximately 5.0 × 10^5^ hiPSCs were seeded per well in a Matrigel‐coated 6‐well plate. After 2 days, pPB and mPB plasmids were co‐transfected as follows. In a 1.5 mL tube A (polyethyleneimine [PEI]), 3 µL PEI MAX solution (1 µg µL^−1^, Polysciences, #24765) was mixed with 47 µL of 150 mM NaCl. In another 1.5 mL tube B (for plasmids), 0.9 µg of pPB and 0.1 µg mPB plasmids (total 1 µg) were added into 49 µL of 150 mM NaCl. After adding the PEI solution to the diluted plasmids, the mixture was incubated for 5 min at room temperature and then dropped onto the *NF2^−/−^* 1 hiPSCs. The medium was replaced 24 h post‐transfection and supplemented with 100 µg mL^−1^ of Hygromycin B Gold (InvivoGen, #ant‐hg‐1). After 2 days, hygromycin‐resistant colonies were replated and expanded under continued hygromycin selection. In the second stage, a Dox‐dependent *NF2* cassette was introduced by lentiviral transduction. The eGFP‐Merlin cassette was isolated from a pEGFP‐Merlin plasmid (Addgene, #84293) and inserted into the NheI (NEB, #R3131)/BamHI (NEB, #R0136) restriction enzyme cutting sites of the FUW‐tetO‐MCS plasmid (Addgene, #84008). 5 µg of the FUW lentiviral vector with 2.5 µg of pMD2.G and 2.5 µg of psPAX3 (both gift from Didier Trono) plasmids were co‐transfected into HEK 293T cells using PEI in a 100 mm cell culture dish. The medium was replaced after 6 h, and the viral supernatant was collected after an additional 48 h of incubation. The collected lentiviral supernatant was filtered using a 0.45 µm polyether sulfone syringe filter and then concentrated with a lentiviral concentrator solution (40% W/V PEG‐800 and 1.2 M NaCl). The mixture was incubated for 1 h at 4 °C and then collected by centrifugation (3000 g, 4 °C, 1 h). The concentrated viruses were resuspended in 1 mL of PBS. The rtTA‐integrated hiPSCs were infected with 1 µL of polybrene (8 µg µL^−1^) and 1 µL of concentrated viral supernatant (MOI: ≈1) in a well of a Matrigel‐coated 6‐well plate. After 48 h, the lentivirus‐infected cells were passaged into a 6‐well plate at a 1:6 ratio. The eGFP‐positive hiPSC clones were recovered by limiting dilution in the presence of doxycycline (1 µg mL^−1^; Sigma #D9891). After selecting stably transfected cells with hygromycin, induced *NF2* expression was analyzed. A Dox concentration of 0.5 µg mL^−1^ was chosen for the rest of the experiments because it secured significant eGFP‐NF2 expression without cytotoxicity.

### hiPSC Culture

Cells were used before passage number 50 and were maintained on Matrigel hESC‐qualified matrix (Corning, #354277) or Geltrex (Gibco, #A1413302) in mTeSR‐1 (STEMCELL Technologies, #85 850) supplemented with 1x penicillin‐streptomycin (Gibco, #15140122). Cells were passaged at ≈80% confluency and hiPSC colonies were treated with 0.5 mM EDTA (Boston BioProducts Inc.) or ReLeSR (StemCell Technologies, #100‐0483) for detaching by tapping the side of the plates. Detached cell clumps were plated on new coated 6‐well plates. The medium was replenished every day.

### Karyotyping and Mycoplasma Testing

Karyotyping was processed by WiCell according to the International System for Human Cytogenetic Nomenclature. The cell lines were evaluated at passage number 5, with 20 cells in metaphase counted for the analysis. A mycoplasma test was performed using the MycoAlert Mycoplasma Detection Kit (Lonza) to ensure all cells were mycoplasma‐free.

### Monolayer Differentiation

For directed differentiation toward ectoderm, mesoderm, and endoderm lineage, STEMdiff Trilineage Differentiation Kit (StemCell Technologies, #05230) was used according to the manufacturer's instructions. It takes 7 days for ectoderm and 5 days for mesoderm and endoderm differentiation.

### EB Formation Procedures

When analyzing endoderm restoration, i*NF2* ^−/−^ hiPSCs were dissociated with ReLeSR and were distributed 3.5 × 10^4^ cells per well onto low‐adhesion 96‐well U‐bottom plates (Millipore Sigma, #CLS7007) in DMEM/F12 (Gibco, #11330057) supplemented with 10% Knockout Serum Replacement and 10 µM Y‐27632 (TOCRIS, #1254). After seeding the cells, the 96‐well plates were centrifuged at low speed (200 g for 3 min). 0.5 µg mL^−1^ of Dox was applied for the first 4 days and half of the medium was replaced every other day until day 20.

### Whole Genome Sequencing of hiPSCs and Off‐Target Detection

Libraries from 2 hiPSC cell lines, WT and *NF2* ^−/^ *^−^* 1 (hiPSC line with *NF2* gene exon 2–4 biallelic excision via CRISPR/Cas9), were prepared. Indexed libraries were pooled and run on the Illumina HiSeq platform as paired‐end 2 × 150 bp runs. Alignment and variant calling were provided by the Harvard Chan Bioinformatics Core (Harvard T.H. Chan School of Public Health, Boston, MA, USA) using an analysis pipeline based on the bcbio framework (https://github.com/bcbio/bcbio‐nextgen). For WGS data, BWA (v0.7.17) was used to map sequencing reads to the reference human genome (hg38). Single nucleotide polymorphisms and indels were called using mutect2 with a somatic tumor‐normal approach (using a control sample as a normal and edited sample as a “tumor”). Structural variants were called Manta (v1.6.0)^[^ ^57^ ^]^ and LUMPY (v0.2.13) (https://github.com/arq5x/lumpy‐sv).^[^ ^58^ ^]^ Variant annotation was performed with Ensembl Variant Effect Predictor (https://useast.ensembl.org/info/docs/tools/vep/index.html) and snpEFF (v4.3T).

Potential off‐targets were identified with Cas‐OFFinder (v2.4), using an offline version allowing up to 7 mismatches and non‐canonical protospacer adjacent motifs (PAMs). The output of sites with at most 6 mismatches in the spacer and at most 1 mismatch in the PAM was restricted. On‐target variants were retrieved from vcfs by filtering all off‐target variants.

### Fluorescence In Situ Hybridization (FISH)

For validation of *NF2* expression, hiPSCs were seeded onto Matrigel‐coated cover glass and fixed with 4% paraformaldehyde (PFA; ThermoFisher Scientific) at ≈80% confluency. FISH was performed using BaseScope Duplex Reagents Kit (Advanced Cell Diagnostics, #323800) and a customized probe (Advanced Cell Diagnostics) according to the manufacturer's instructions. Following FISH, fluorescence images were acquired with a confocal microscope (Zeiss LSM 880) and processed in the same protocol (maximum intensity projection, 8‐bit image conversion, threshold set as “Moments”, *n* = 4, duplicated). Subsequently, fluorescence signals were quantified by counting the particles using Fiji software^[^ ^59^ ^]^ with the “Analyze particles” plugin.

### Endoderm Differentiation Procedures

To induce endoderm differentiation for Figures 3, 4, 5, 6, a modified published protocol was used.^[^ ^60^ ^]^ hiPSCs were passaged on Geltrex in mTeSR‐1 with 10 µM of Y‐27632 for 24 h at 1.0 × 10^5^ cells cm^2^. On day 1, the medium was replaced with RPMI‐1640 (Gibco, #11875093) supplemented with GlutaMAX (Gibco, # 35050061), 100 ng mL^−1^ Activin A (R&D SYSTEMS, 338‐AC‐010), 1x B27 (Gibco, #17504‐044), and 2.5 µM of CHIR99021 (TOCRIS, #4423). On day 2, the medium was again replaced with RPMI‐1640 containing GlutaMAX, 100 ng mL^−1^ Activin A, and 0.2% Knockout Serum Replacement (Gibco, #10828028). On day 3, the medium was replaced with RPMI‐1640 containing GlutaMAX,100 ng mL^−1^ Activin A, and 2% Knockout Serum Replacement. The differentiated cells were used for experiments on days 4 or 7.

### Knockdown of *YAP1/TAZ* with siRNA

For the siRNA knockdown studies, cells were transfected with 5 pmole (10 nM) of *YAP1* (Ambion, #107951, #107952) or *TAZ* siRNA (Ambion, #122501, #122502) with Lipofectamine RNAiMAX Transfection reagent (Invitrogen, #13778100) during endoderm differentiation. Negative Control #1 siRNA (Ambion, #AM4611), which was designed to have sequences that do not target any gene product, was used as the control. siRNAs were diluted in Opti‐MEM I Reduced Serum Medium (Gibco, #31985062) and incubated with the transfection reagent for 5 min at room temperature before treatment. The duration of *YAP1* siRNA application and the volume of the transfection reagent were optimized. The treatment duration of days 0–1 and a volume of 1.5 µL RNAiMAX were selected as the best conditions to achieve maximal restoration of the expression of endoderm markers with minimal reagents (Figure S4A,B, Supporting Information). For decoupling YAP1 and TEAD, 62.5 and 125 nM of verteporfin (MedChemExpress, #HY‐B0146) were treated between days 0 and 1 during endoderm differentiation.

### Inhibition of the TGFβ and BMP Pathways

For blocking the TGFβ and BMP signaling pathways, a final concentration of 1 µM SB431542 (TOCRIS, #1614) and 100 nM LDN193189 (Stemgent, #04‐0074‐02), respectively, were applied during day 0–4 of endoderm differentiation. For the negative control, the same volume of solvent DMSO (Sigma, #D2650) was used.

### RT‐qPCR Analysis

Total RNA was isolated using RNeasy Mini Kits (Qiagen, #74104), and 0.5 µg or 1 µg was used for cDNA synthesis. A reverse transcription reaction was performed using SuperScript IV (Invitrogen, #18090050) including oligo dT according to the manufacturer's instructions. RT‐qPCR was performed in a QuantStudio 6 Pro Real‐Time PCR system by using a reaction mixture with GoTaq qPCR Master Mix (Promega, #A6002). The primers used are listed in Table S3 (Supplementary Table). The PCR cycle parameters were 50 °C for 2 min, 95 °C for 10 min, 40 cycles with denaturation at 95 °C for 15 s and annealing at 60 °C for 1 min. All gene expression experiments were performed at least 3 times, each time in triplicate. The expression levels were normalized to that of *GAPDH* and were displayed in arbitrary units, 2^−ΔΔCt^.

### RNA Sequencing

All the hiPSCs were harvested at ≈80% confluency and total RNA was isolated. i*NF2* ^−/−^ hiPSCs were treated with 0.5 µg mL^−1^ Dox for 48 h prior to differentiation and during 4 days of endoderm differentiation to induce *NF2* expression. The quality of RNA samples was checked and met the criteria of purity (A260/280) between 1.8 and 2.2 and RNA integrity number above 6. The RNA samples were subjected to poly(A)+ selection, cDNA synthesis, and sequencing using Illumina HiSeq in GENEWIZ (https://www.genewiz.com/), resulting in 150‐nt paired‐end reads per sample. RNA sequencing sample quality control and statistics information are available in Table S5 (Supplementary Table).

### Gene Set Enrichment Analysis

DEGs were identified for hiPSC state using the DESeq2 package^[^ ^61^ ^]^ (version 1.42.1) in R (version 4.3.3). Normalized count data were filtered based on an adjusted *p* < 0.05 and a |log_2_(fold‐change)| > 1 threshold to determine significant DEGs. GO terms and corresponding gene annotations were obtained using the GO.db package (version 3.18.0). The results of the GO analysis were visualized using the ComplexHeatmap package^[^ ^62^ ^]^ (version 2.18.0).

|log_2_(fold‐change)| > 1 DEGs in *NF2* ^−/−^ 1 compared to WT hiPSCs after endoderm differentiation (Table S2, Supplementary Table) were used as the input for gene set enrichment analysis using Cytoscape and its plugin Bingo. The most recent GO file “go‐basic.obo” was downloaded from the Gene Ontology Consortium website (http://geneontology.org/) instead of using Bingo's default ontologies. Significant GO terms (adjusted *p <* 0.05) were used as input for Cytoscape's plugin Enrichment Map to generate a network.

### Western Blot Procedures

Cells were seeded into a 6‐well plate, cultured for 2 days in the absence or presence of Dox, and then lysed in RIPA buffer (Thermo Scientific, #89900) containing 1x protease/phosphatase inhibitors (Thermo Fisher Scientific, #32959). The samples were centrifuged at 12000 g for 10 min at 4 °C, then the supernatants were transferred to fresh 1.5 mL tubes. The protein concentrations were measured using the BCA protein assay kit (Thermo Fisher Scientific, #23225) and the lysates were boiled at 95 °C for 5 min. 30 µg of lysates were loaded on Bolt 4% – 12% Bis‐Tris Plus gels (Thermo Fisher Scientific, #NW04120BOX). After separation, proteins were transferred onto Low Fluorescence PVDF membranes (Azure Biosystems, #AC2105). Membranes were blocked with blocking buffer (Azure Biosystems, #AC2190) for 1 h at room temperature and incubated with primary antibodies overnight at 4 °C. Membranes were washed and incubated with secondary antibodies for 1 h at room temperature in the blocking buffer. Chemiluminescent and fluorescence blotting images were developed using the Azure600 system. See Table S6 (Supplementary Table) for the list of antibodies.

### Immunostaining Procedures

For immunocytochemistry, cells grown on coverslips were fixed with 4% PFA for 10 min at room temperature. For permeabilization, cells were washed 3 times with Ca^2+^/Mg^2+^ PBS (Gibco, #14040117) and incubated in PBST, which was 0.1% Triton X‐100 (Sigma, #X100) in 1x PBS solution, for 10 min at room temperature. Unspecific binding was blocked with 5% normal horse serum (Abcam, #ab139501) or 5% goat serum (Gibco, #16210064) in PBST for 1 h. Samples were then incubated overnight at 4 °C with specific primary antibodies (Table S6, Supplementary Table) diluted in 1% BSA (Sigma, #A8412) in PBST, washed 3 times with PBS, and incubated with secondary antibodies (Table S6, Supplementary Table) in PBST. For F‐actin staining, Rhodamine Phalloidin (Cytoskeleton, #PHDR1) at 1:500 dilution was used.

For immunohistochemistry, EBs were fixed with 4% PFA for 20 min at room temperature. The fixed specimens were cryopreserved with a graded treatment of 10%, 20%, and 30% sucrose (Sigma, #S9378) overnight at 4 °C and then embedded in tissue freezing medium OCT compound (Fisher HealthCare, #4583). Frozen tissue blocks were sectioned into 12 µm cryosections on a Leica CM‐1860 cryostat. For immunostaining, a 5% goat or horse serum in 0.3% Triton X‐100 1x PBS solution was used for blocking, and a 1% BSA and 0.3% Triton X‐100 1x PBS solution was used for primary and secondary antibody (Table S6, Supplementary Table) incubations.

Vectashield (Vector Laboratories, #H‐1000‐10) with 1x DAPI (Cell Signaling, #4083S) was used to mount the samples and visualize cellular nuclei. Negative control experiments without the primary antibodies were processed in parallel. Microscopy was performed using a Leica SP8 confocal microscope (Leica Microsystems) or Zeiss LSM 880.

### In Vivo Teratoma Formation Assay

All animal experiments were performed by a genome modification facility at Harvard University. Three mice aged 6–7 weeks (NOD.CB17‐Prkdc<scid>/J, strain 001303) were used to test the in vivo proliferative capacity of the *NF2^−/−^* 1 (*n* = 3 mice) and WT (*n* = 3 mice) hiPSC lines. Ten to 15 clumps of 200–400 cells per clump of WT and *NF2^−/−^* 1 hiPSCs were injected into the kidney capsule as previously described.^[^ ^63^ ^]^ 8 to 11 weeks following implantation, the mice were sacrificed and the teratomas were isolated and fixed with 4% PFA for 24 h. To measure the size of teratomas, stereomicroscopic images of the teratomas were imported into Fiji software (ImageJ version 1.54i). The scale of the image was established using a one‐cent coin placed adjacent to the teratomas in the field of view. Teratomas were manually outlined around the area of each sample using the “Polygon Selection” tool. The area of each selected region was measured using the “Analyze → Measure” function. The provided area in square pixels was converted to square millimeters using the established cent coin calibration. Statistical analysis of teratoma size measurements was performed using a *t*‐test in the GraphPad Prism software (version 9.5).

For histological analysis, the teratomas were embedded in paraffin wax, sectioned at 5 µm, and then analyzed by hematoxylin and eosin staining. Further details are available from the genome modification facility (https://gmf.fas.harvard.edu/pages/contact‐1).

### Quantification and Statistical Analysis

The representative phase contrast images shown in Figures 1A and 5B represent at least 3 different passage numbers of cells. The representative phase contrast images shown in Figures 3C and 4D represent at least 3 independent experiments.

Bar graphs in Figures 1, 2, 3, 4, 5F show at least 3 biological replicates with 2–3 technical replicates for each cell line or each condition. FISH of fluorescent images in Figure 1C,C’ are representative of 4 independent experiments with 2 technical replicates. All immunostaining images (Figures 1, 2, 3, 4, 5, 6) shown in this article are representative of a minimum of 3 independent experiments with 2–4 replicates. RNA sequencing data shown in Figures 2, 5, 6B–D, and Western blot results shown in Figures 3F and 5C were generated from 3 independent experiments with 3 different passage numbers of cell lines. In the comparison of cell thickness using a HoloMonitor shown in Figures 2D and 3 different passage numbers of cells were used and at least 3 colonies were measured.

For experiments using the Dox‐inducible system, 3 clones were generated and tested. Clone 3 (i*NF2* ^−/−^) was chosen for further experiments shown in Figures 5, 6. Western blot for eGFP‐NF2 and GAPDH was performed at least 4 times (Figure 5C). When measuring EB size and eccentricity, day 20 and 15 EB were used, respectively, from at least 3 independent experiments (Figure 6F).

Statistical significance was determined using unpaired *t*‐test for Figures 1, 3, 4, 5, 6F using Prism or the rstatix package (version 0.7.2) in R. Statistical analyses for Figure 2E were performed with one‐way ANOVA using Prism. The Benjamini and Hochberg method was used to calculate *p*‐values corrected for multiple comparisons (adjusted *p*). Statistical significance was determined using *p *< 0.05 for all analyses (adjusted and unadjusted).

For the teratoma histology analysis in Figure 1G, 5 images were taken from each mouse. Semi‐quantitative analyses for Figure 5G, 6A; Figure S5E (Supporting Information) are provided in Table S7 (Supplementary Table).

All experiments described in this study were replicated by 2 independent investigators in separate locations (M.J. [California] and D.H [Massachusetts and California]) using the independently generated *NF2^−/−^* cell lines *NF2^−/−^* 1 and *NF2^−/−^* 2. Thus, the success of reproducibility was confirmed that not limited by a person, cell lines, or place.

### Ethics Approval Statement and Informed Consent

The protocols for research involving tissues from human subjects and for stem cell research were approved by the institutional review board of Massachusetts Eye and Ear and Partners Human Research Committee (to K.M.S; date 05/25/2017). All study participants provided written informed consent. The protocols for research involving mice were approved by MEE Human Studies Committee, Boston, MA (14‐148H, 10/22/2019).

## Conflict of Interest

The authors declare no conflict of interest.

## Author Contributions

M.J. and D.H. contributed equally to this work. M.J. and D.H. conceived, designed, and led the study, performed experiments, analyzed data, and wrote the manuscript. M.J. drafted the manuscript with input from all authors. P.B. performed bioinformatic analysis and critically reviewed the manuscript. D.B.W. consented to patients for the skin biopsy for hiPSC generation and critically reviewed the manuscript. M.S. guided in vivo experiments and critically reviewed the manuscript. K.M.S. conceived, designed, and led the study, critically analyzed data, supervised all aspects of research, and edited the manuscript.

## Supporting information



Supporting Information

Supplementary Table 1

Supplementary Table 2

Supplementary Table 3

Supplementary Table 4

Supplementary Table 5

Supplementary Table 6

Supplementary Table 7

## Data Availability

All source data supporting this study are available within the paper and its Supplementary Figures and Tables. Bulk RNA sequencing data have been deposited in Sequence Read Archive (SRA) and are publicly available as of the date of publication (SRA accession number: PRJNA1141571). The codes for RNA sequencing analysis are available on the following website: https://github.com/djhan0110/NF2_endoderm. Additional data are available from the corresponding author upon reasonable request.

## References

[1] S. Menchero , T. Rayon , M. J. Andreu , M. Manzanares , Dev. Dyn. 2017, 246, 245. 27859869 10.1002/dvdy.24471

[2] K. Cockburn , S. Biechele , J. Garner , J. Rossant , Curr. Biol. 2013, 23, 1195. 23791728 10.1016/j.cub.2013.05.044

[3] a) M. Mota , L. A. Shevde , Cell Commun. Signal 2020, 18, 63; 32299434 10.1186/s12964-020-00544-7 PMC7164249

[4] J. Huang , S. Wu , J. Barrera , K. Matthews , D. Pan , Cell 2005, 122, 421. 16096061 10.1016/j.cell.2005.06.007

[5] Z. Wu , K. L. Guan , Trends Biochem. Sci. 2021, 46, 51. 32928629 10.1016/j.tibs.2020.08.008 PMC7749079

[6] a) P. Li , Y. Chen , K. K. Mak , C. K. Wong , C. C. Wang , P. Yuan , PLoS One 2013, 8, e79867; 24224013 10.1371/journal.pone.0079867 PMC3818222

[7] a) Z. Chen , G. A. Friedrich , P. Soriano , Genes Dev. 1994, 8, 2293; 7958896 10.1101/gad.8.19.2293

[8] a) K. Zeevaert , R. Goetzke , M. H. Elsafi Mabrouk , M. Schmidt , C. Maassen , A. C. Henneke , C. He , A. Gillner , M. Zenke , W. Wagner , Biomater. Adv. 2023, 146, 213308; 36774716 10.1016/j.bioadv.2023.213308

[9] M. Zhou , P. Huang , R. Bai , X. Liu , Stem Cell Res. 2022, 64, 102923. 36219982 10.1016/j.scr.2022.102923

[10] a) M. Giovannini , E. Robanus‐Maandag , M. van der Valk , M. Niwa‐Kawakita , V. Abramowski , L. Goutebroze , J. M. Woodruff , A. Berns , G. Thomas , Genes Dev. 2000, 14, 1617; 10887156 PMC316733

[11] a) M. E. Baser , L. Kuramoto , R. Woods , H. Joe , J. M. Friedman , A. J. Wallace , R. T. Ramsden , S. Olschwang , E. Bijlsma , M. Kalamarides , L. Papi , R. Kato , J. Carroll , C. Lázaro , F. Joncourt , D. M. Parry , G. A. Rouleau , D. G. Evans , J. Med. Genet. 2005, 42, 540; 15994874 10.1136/jmg.2004.029504 PMC1736092

[12] H. Hentze , P. L. Soong , S. T. Wang , B. W. Phillips , T. C. Putti , N. R. Dunn , Stem Cell Res. 2009, 2, 198. 19393593 10.1016/j.scr.2009.02.002

[13] D. Gökbuget , R. Blelloch , Development 2019, 146, F542. 10.1242/dev.164772 PMC6803368 31554624

[14] R. A. Young , Cell 2011, 144, 940. 21414485 10.1016/j.cell.2011.01.032 PMC3099475

[15] A. V. A. Mariadoss , C. Z. Wang , Int. J. Mol. Sci. 2023, 24.

[16] M. Curto , A. I. McClatchey , Br. J. Cancer 2008, 98, 256. 17971776 10.1038/sj.bjc.6604002 PMC2361439

[17] T. Vessella , S. Xiang , C. Xiao , M. Stilwell , J. Fok , J. Shohet , E. Rozen , H. S. Zhou , Q. Wen , FEBS Open Bio 2024, 14, 867. 10.1002/2211-5463.13798 PMC11073507 38538106

[18] S. Liu , P. Kanchanawong , J. Cell Sci. 2022, 135, 14895. 10.1242/jcs.259379 35726598

[19] A. Menke , C. Philippi , R. Vogelmann , B. Seidel , M. P. Lutz , G. Adler , D. Wedlich , Cancer Res. 2001, 61, 3508. 11309315

[20] a) D. Buehler , H. Hardin , W. Shan , C. Montemayor‐Garcia , P. S. Rush , S. Asioli , H. Chen , R. V. Lloyd , Mod. Pathol. 2013, 26, 54; 22899291 10.1038/modpathol.2012.137 PMC3559085

[21] K. Burridge , K. Fath , T. Kelly , G. Nuckolls , C. Turner , Annu. Rev. Cell Biol. 1988, 4, 487. 3058164 10.1146/annurev.cb.04.110188.002415

[22] a) G. Nardone , J. Oliver‐De La Cruz , J. Vrbsky , C. Martini , J. Pribyl , P. Skládal , M. Pešl , G. Caluori , S. Pagliari , F. Martino , Z. Maceckova , M. Hajduch , A. Sanz‐Garcia , N. M. Pugno , G. B. Stokin , G. Forte , Nat. Commun. 2017, 8, 15321; 28504269 10.1038/ncomms15321 PMC5440673

[23] K. Striedinger , S. R. VandenBerg , G. S. Baia , M. W. McDermott , D. H. Gutmann , A. Lal , Neoplasia 2008, 10, 1204. 18953429 10.1593/neo.08642 PMC2570596

[24] B. Zhao , X. Wei , W. Li , R. S. Udan , Q. Yang , J. Kim , J. Xie , T. Ikenoue , J. Yu , L. Li , P. Zheng , K. Ye , A. Chinnaiyan , G. Halder , Z. C. Lai , K. L. Guan , Genes Dev. 2007, 21, 2747. 17974916 10.1101/gad.1602907 PMC2045129

[25] F. Reggiani , G. Gobbi , A. Ciarrocchi , V. Sancisi , Trends Biochem. Sci. 2021, 46, 154. 32981815 10.1016/j.tibs.2020.08.012

[26] A. K. Teo , S. J. Arnold , M. W. Trotter , S. Brown , L. T. Ang , Z. Chng , E. J. Robertson , N. R. Dunn , L. Vallier , Genes Dev. 2011, 25, 238. 21245162 10.1101/gad.607311 PMC3034899

[27] Y. Liu‐Chittenden , B. Huang , J. S. Shim , Q. Chen , S. J. Lee , R. A. Anders , J. O. Liu , D. Pan , Genes Dev. 2012, 26, 1300. 22677547 10.1101/gad.192856.112 PMC3387657

[28] N. S. Funa , K. A. Schachter , M. Lerdrup , J. Ekberg , K. Hess , N. Dietrich , C. Honoré , K. Hansen , H. Semb , Cell Stem Cell 2015, 16, 639. 25921273 10.1016/j.stem.2015.03.008

[29] I. Darby , O. Skalli , G. Gabbiani , Lab. Invest. 1990, 63, 21. 2197503

[30] G. Gabbiani , G. B. Ryan , G. Majne , Experientia 1971, 27, 549. 5132594 10.1007/BF02147594

[31] M. Otranto , V. Sarrazy , F. Bonté , B. Hinz , G. Gabbiani , A. Desmoulière , Cell Adh. Migr. 2012, 6, 203. 22568985 10.4161/cam.20377 PMC3427235

[32] A. Biernacka , M. Dobaczewski , N. G. Frangogiannis , Groundwater 2011, 29, 196. 10.3109/08977194.2011.595714 PMC4408550 21740331

[33] a) B. Herrera , A. Addante , A. Sánchez , Int. J. Mol. Sci. 2017, 19; 10.3390/ijms19010039 PMC5795989 29295498

[34] T. C. Doetschman , H. Eistetter , M. Katz , W. Schmidt , R. Kemler , J. Embryol. Exp. Morphol. 1985, 87, 27. 3897439

[35] K. Zeevaert , M. H. Elsafi Mabrouk , W. Wagner , R. Goetzke , Cells 2020, 9. 10.3390/cells9102270 PMC7599659 33050550

[36] X. Zhu , Z. Min , R. Tan , Q. Tao , Mech. Dev. 2015, 138, 305. 26344136 10.1016/j.mod.2015.08.008

[37] A. M. Petrilli , C. Fernández‐Valle , Oncogene 2016, 35, 537. 25893302 10.1038/onc.2015.125 PMC4615258

[38] H. T. Hsu , C. Estarás , L. Huang , K. A. Jones , Stem Cell Rep. 2018, 11, 1357. 10.1016/j.stemcr.2018.10.013 PMC6294113 30449705

[39] A. Nourbakhsh , N. C. Gosstola , C. Fernandez‐Valle , D. M. Dykxhoorn , X. Z. Liu , Stem Cell Res. 2021, 55, 102474. 34352618 10.1016/j.scr.2021.102474

[40] Y. Ishi , T. Era , S. Yuzawa , M. Okamoto , R. Sawaya , H. Motegi , S. Yamaguchi , S. Terasaka , K. Houkin , M. Fujimura , Am. J. Med. Genet. A 2022, 188, 1863. 35178855 10.1002/ajmg.a.62700

[41] N. Catasús , M. Torres‐Martin , A. Negro , B. Kuebler , I. Rosas , G. Casals , H. Mazuelas , F. Roca‐Ribas , E. Amilibia , B. Aran , A. Veiga , Á. Raya , B. Gel , I. Blanco , E. Serra , M. Carrió , E. Castellanos , bioRxiv 2022, 2022.

[42] J. S. Mo , H. W. Park , K. L. Guan , EMBO Rep. 2014, 15, 642. 24825474 10.15252/embr.201438638 PMC4197875

[43] R. Ebrahimighaei , G. B. Sala‐Newby , C. Hudson , T. E. Kimura , T. Hathway , J. Hawkins , M. C. McNeill , R. Richardson , A. C. Newby , M. Bond , Biochimica et Biophysica Acta (BBA) – Mol. Cell Res. 2022, 1869, 119329. 10.1016/j.bbamcr.2022.119329 PMC7616274 35905788

[44] T. A. Beyer , M. Narimatsu , A. Weiss , L. David , J. L. Wrana , Biochim. Biophys. Acta 2013, 1830, 2268. 22967760 10.1016/j.bbagen.2012.08.025

[45] L. Vallier , S. Mendjan , S. Brown , Z. Chng , A. Teo , L. E. Smithers , M. W. Trotter , C. H. Cho , A. Martinez , P. Rugg‐Gunn , G. Brons , R. A. Pedersen , Development 2009, 136, 1339. 19279133 10.1242/dev.033951 PMC2687465

[46] a) X. Varelas , R. Sakuma , P. Samavarchi‐Tehrani , R. Peerani , B. M. Rao , J. Dembowy , M. B. Yaffe , P. W. Zandstra , J. L. Wrana , Nat. Cell Biol. 2008, 10, 837; 18568018 10.1038/ncb1748

[47] C. E. Murry , G. Keller , Cell 2008, 132, 661. 18295582 10.1016/j.cell.2008.02.008

[48] A. S. Bernardo , T. Faial , L. Gardner , K. K. Niakan , D. Ortmann , C. E. Senner , E. M. Callery , M. W. Trotter , M. Hemberger , J. C. Smith , L. Bardwell , A. Moffett , R. A. Pedersen , Cell Stem Cell 2011, 9, 144. 21816365 10.1016/j.stem.2011.06.015 PMC3567433

[49] F. L. Conlon , K. M. Lyons , N. Takaesu , K. S. Barth , A. Kispert , B. Herrmann , E. J. Robertson , Development 1994, 120, 1919. 7924997 10.1242/dev.120.7.1919

[50] a) S. Lee , P. J. Karas , C. C. Hadley , V. J. Bayley , A. B. Khan , A. Jalali , A. D. Sweeney , T. J. Klisch , A. J. Patel , Cancers 2019, 11; 10.3390/cancers11111633 PMC6893739 31652973

[51] a) K. Hess , D. C. Spille , A. Adeli , P. B. Sporns , K. Zitta , L. Hummitzsch , J. Pfarr , W. Stummer , B. Brokinkel , R. Berndt , M. Albrecht , Cancers 2020, 12; 10.3390/cancers12103075 PMC7593950 33096816

[52] M. M. Mia , M. K. Singh , Cells 2022, 11. 10.3390/cells11132065 PMC9265296 35805148

[53] B. Piersma , S. de Rond , P. M. N. Werker , S. Boo , B. Hinz , M. M. van Beuge , R. A. Bank , Am. J. Path. 2015, 185, 3326. 26458763 10.1016/j.ajpath.2015.08.011

[54] F. Calvo , N. Ege , A. Grande‐Garcia , S. Hooper , R. P. Jenkins , S. I. Chaudhry , K. Harrington , P. Williamson , E. Moeendarbary , G. Charras , E. Sahai , Nat. Cell Biol. 2013, 15, 637. 23708000 10.1038/ncb2756 PMC3836234

[55] M. E. Sughrue , R. Kaur , A. J. Kane , M. J. Rutkowski , I. Yang , L. H. Pitts , T. Tihan , A. T. Parsa , J. Neurosurg. 2011, 114, 386. 20560722 10.3171/2010.5.JNS10256

[56] a) J. Cadiñanos , A. Bradley , Nucleic Acids Res. 2007, 35, e87; 17576687 10.1093/nar/gkm446 PMC1919496

[57] X. Chen , O. Schulz‐Trieglaff , R. Shaw , B. Barnes , F. Schlesinger , M. Källberg , A. J. Cox , S. Kruglyak , C. T. Saunders , Bioinformatics 2016, 32, 1220. 26647377 10.1093/bioinformatics/btv710

[58] R. M. Layer , C. Chiang , A. R. Quinlan , I. M. Hall , Genome Biol. 2014, 15, R84. 24970577 10.1186/gb-2014-15-6-r84 PMC4197822

[59] J. Schindelin , I. Arganda‐Carreras , E. Frise , V. Kaynig , M. Longair , T. Pietzsch , S. Preibisch , C. Rueden , S. Saalfeld , B. Schmid , J. Y. Tinevez , D. J. White , V. Hartenstein , K. Eliceiri , P. Tomancak , A. Cardona , Nat. Methods 2012, 9, 676. 22743772 10.1038/nmeth.2019 PMC3855844

[60] J. Saari , F. Siddique , S. Korpela , E. Mäntylä , T. O. Ihalainen , K. Kaukinen , K. Aalto‐Setälä , K. Lindfors , K. Juuti‐Uusitalo , Int. J. Mol. Sci. 2022, 23. 10.3390/ijms23031312 PMC8835723 35163236

[61] M. I. Love , W. Huber , S. Anders , Genome Biol. 2014, 15, 550. 25516281 10.1186/s13059-014-0550-8 PMC4302049

[62] Z. Gu , R. Eils , M. Schlesner , Bioinformatics 2016, 32, 2847. 27207943 10.1093/bioinformatics/btw313

[63] R. L. Wesselschmidt , Methods Mol. Biol. 2011, 767, 231. 21822879 10.1007/978-1-61779-201-4_17

