# Supplementary material for: NF2 is Essential for Human Endoderm Development
Source: Adv Sci (Weinh). 2025 Feb 8;12(17):2410909. doi: 10.1002/advs.202410909 (PMC12061267; doi:10.1002/advs.202410909)
Supplement: Supplementary file 1 — Supporting Information [file ADVS-12-2410909-s002.pdf]

ADVANCED SCIENCE
Open Access

Supporting Information

for *Adv. Sci.*, DOI 10.1002/advs.202410909

NF2 is Essential for Human Endoderm Development

*Minjin Jeong, Dongjun Han, Preetida Bhetariya, D. Bradley Welling, Miodrag Stojkovic and Konstantina M. Stankovic**

Supporting Information

NF2 is essential for human endoderm development

*Minjin Jeong, Dongjun Han, Preetida Bhetariya, D. Bradley Welling, Miodrag Stojkovic, Konstantina M. Stankovic**

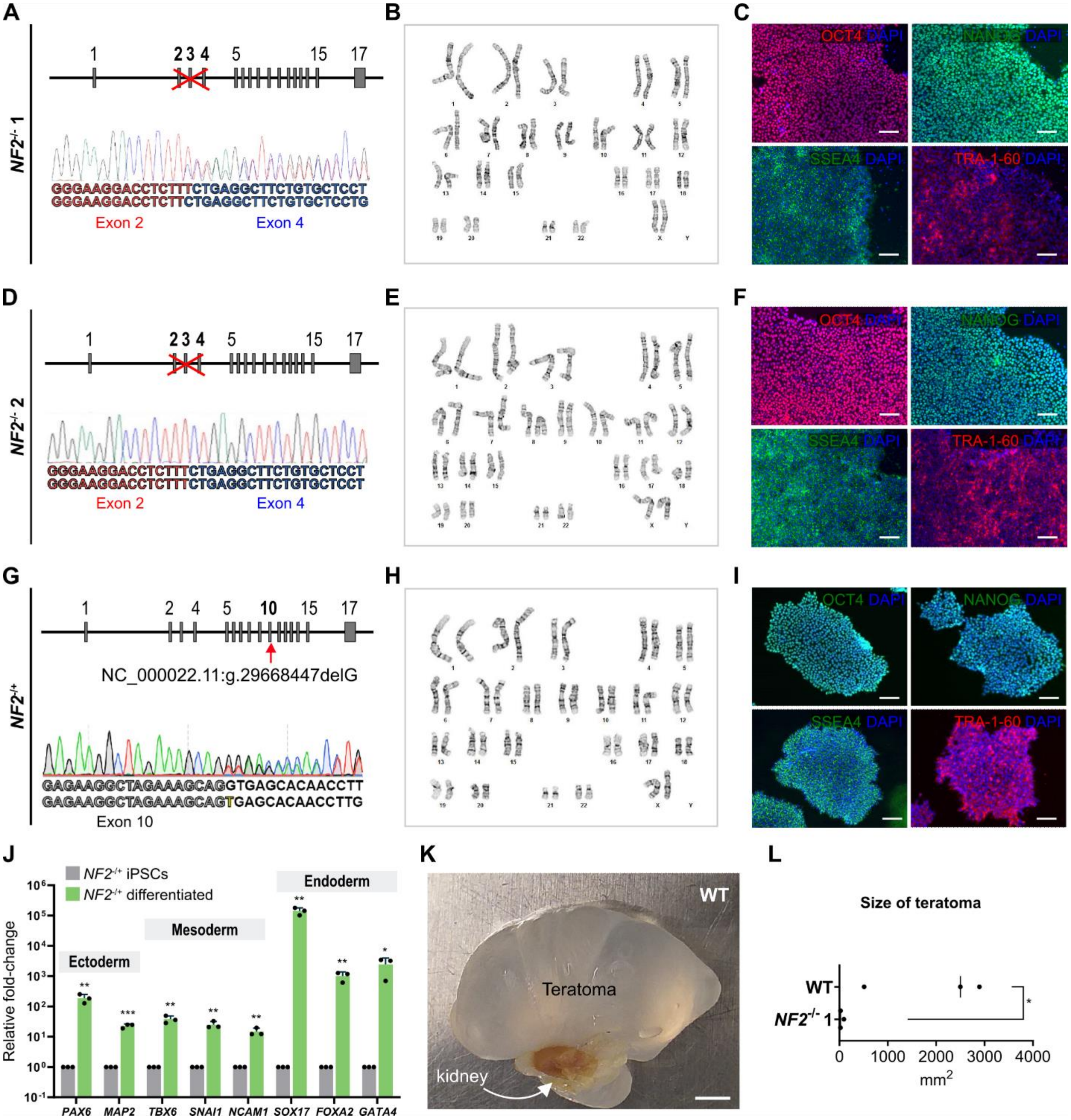

Figure S1. Generation of hiPSCs with homozygous or heterozygous *NF2* deletion, and failure of endoderm formation by *NF2*$^{-/-}$ cells.

A, D) Confirmation of CRISPR/Cas9-mediated biallelic *NF2* exon 2-4 targeting in *NF2*$^{-/-}$ 1 (A) and *NF2*$^{-/-}$ 2 (D) hiPSCs by genomic DNA sequencing. B, E, H) Karyotyping of *NF2*$^{-/-}$ 1 (B), *NF2*$^{-/-}$ 2 (E), and *NF2*$^{-/+}$ (H) hiPSCs showing normal chromosomes. C, F, I) Representative immunofluorescence images of hiPSC colonies showing the presence of pluripotency markers

OCT4, NANOG, SSEA4, and TRA-1-60 in *NF2*$^{-/-}$ 1 (C), *NF2*$^{-/-}$ 2 (F), and *NF2*$^{-/+}$ (I) hiPSCs. Scale bars, 100 μm. G) *NF2* genomic DNA sequencing of *NF2*$^{-/+}$ hiPSCs, derived from a patient with neurofibromatosis type 2 (NF2), showing a heterozygous intronic single nucleotide polymorphism (NC_000022.11:g.29668447delG). J) Tri-lineage differentiation as confirmed by markers of ectoderm (*PAX6*, *MAP2*), mesoderm (*TBX6*, *SNAI1*, *NCAM1*), and endoderm (*SOX17*, *FOXA2*, *GATA4*). Relative fold-changes of expression in differentiated cells from *NF2*$^{-/+}$ hiPSCs demonstrate all tri-lineage cells can be generated from *NF2*$^{-/+}$ hiPSCs. The average expression in *NF2*$^{-/+}$ hiPSCs was set to 1. K) Morphology of the WT hiPSC-injected teratoma. Scale bar, 5 mm. L) Comparison of teratoma size between WT and *NF2*$^{-/-}$ 1. The teratomas arising from WT hiPSCs were significantly larger and had greater capacity for endoderm formation compared with the single teratoma arising from *NF2*$^{-/-}$ 1 hiPSCs injection. $^{*}p < 0.05$, $^{**}p < 0.01$, $^{***}p < 0.001$.

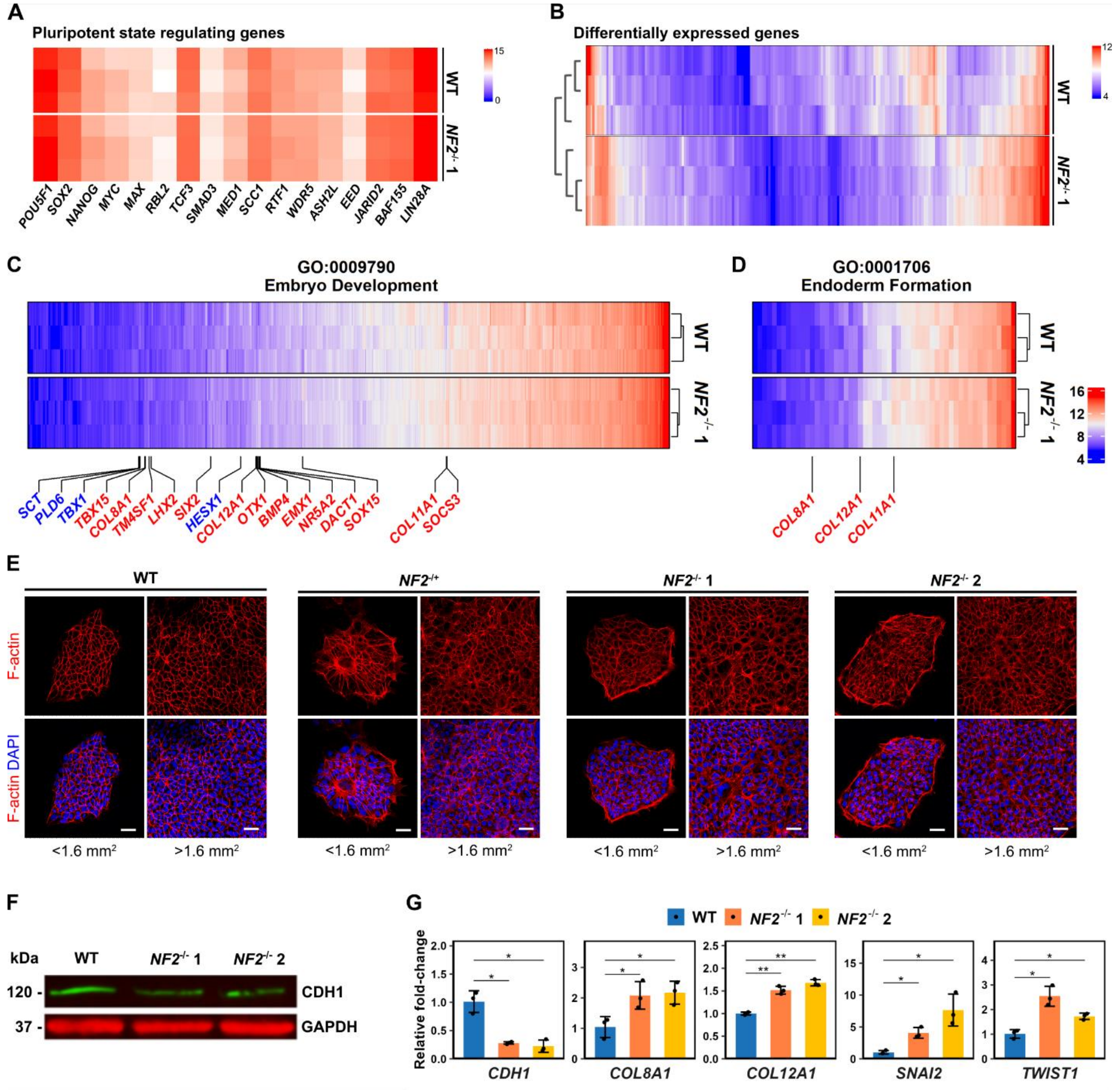

Figure S2. Comparison of transcriptome profiles and F-actin organization between *NF2*-deficient and WT hiPSCs.

A) Heatmap of pluripotent state regulating genes comparing WT and *NF2*$^{-/-}$ 1 hiPSCs. Color denotes normalized gene expression values, with darkest blue as lowest expression and darkest red as highest. B) A clustered heatmap of all differentially expressed genes comparing WT and *NF2*$^{-/-}$ 1 hiPSCs. Color denotes normalized gene expression values, with darkest blue as lowest expression and darkest red as highest. C) Heatmaps of differentially expressed genes (DEGs) in *NF2*$^{-/-}$ 1 hiPSCs associated with selected GO terms. Embryo development (GO:0009790). D)

Endoderm formation (GO:0001706). E) Immunostaining images showing disruption of F-actin structures in *NF2*$^{-/-}$ hiPSCs, but not in WT. Scale bars, 100 μm. F) Western blot of CDH1 expression in WT and *NF2*$^{-/-}$ hiPSCs. G) RT-qPCR analysis of *CDH1* and its regulatory genes. Relative expression levels were normalized to *GAPDH* and presented as fold-changes compared to the WT condition. Bars represent mean±SD fold-change (n = 3, biological replicates). Statistical significance was determined using t-test; $^{*}p < 0.05$, $^{**}p < 0.01$.

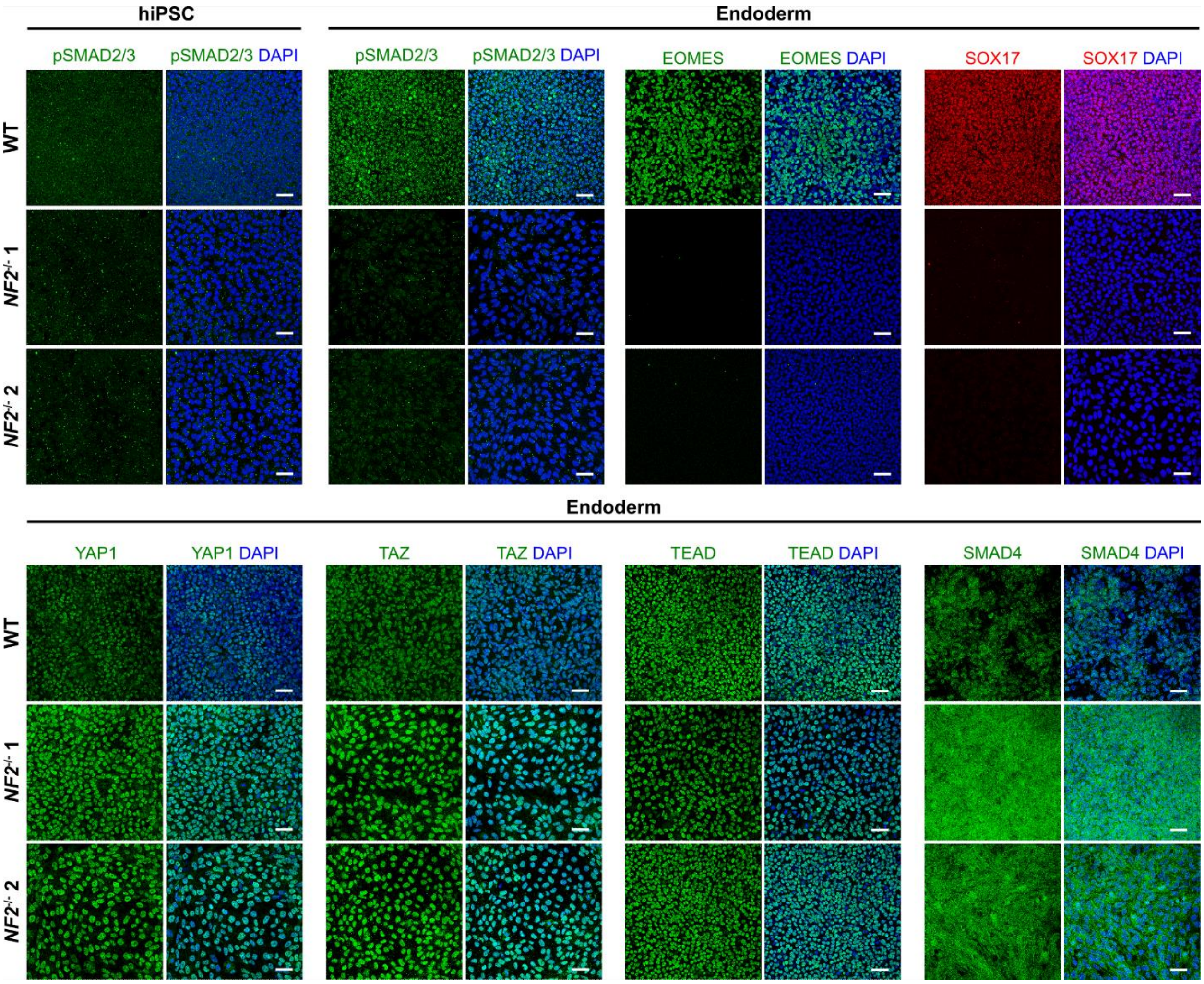

Figure S3. Comparison of YAP/TAZ location and expression of Activin/Nodal pathway proteins.

A) Immunostaining images showing small expression of pSMAD2/3 in WT, *NF2*$^{-/-}$ 1, and *NF2*$^{-/-}$ 2 hiPSCs. Scale bars, 50 µm. B) Immunostaining of pSMAD2/3, EOMES, SOX17, YAP1, TAZ, TEAD, and SMAD4 in WT, *NF2*$^{-/-}$ 1, and *NF2*$^{-/-}$ 2 under endoderm differentiation. Scale bars, 50 µm. All immunostaining images are representative of a minimum of 3 independent experiments with 3 replicates.

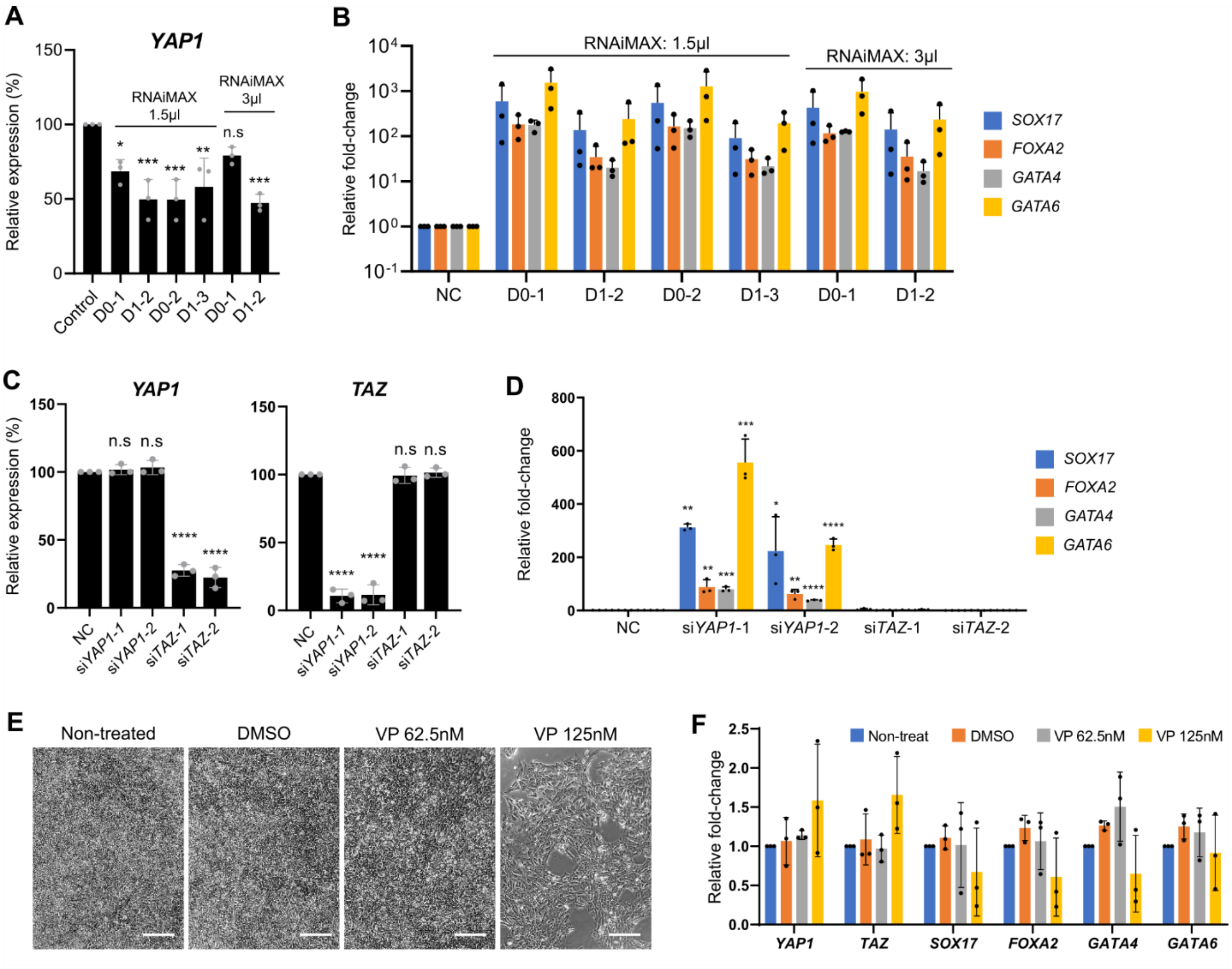

Figure S4. Endoderm differentiation was rescued in *NF2*$^{-/-}$ cells via application of *YAP1* siRNA, but not by *TAZ* siRNA or YAP1 and TEAD decoupling verteporfin.

A, B) Optimization of the duration of *YAP1* siRNA application and the amount of the transfection reagent, RNAiMAX, during endoderm differentiation. *NF2*$^{-/-}$ 1 cells were assessed using the reduction of *YAP1* expression (A) and induction of endoderm markers (B) at the mRNA level on day (D) 4 of endoderm differentiation. A treatment duration of day 0-1 and 1.5 µl of RNAiMAX were selected as the best conditions. C) Relative expressions of *YAP1* and *TAZ* mRNA in *NF2*$^{-/-}$ 2 cells on day 1 of endoderm differentiation after treatment with si*YAP1* or si*TAZ* day 0-1. D) Relative fold-changes of endoderm markers at the mRNA level in *NF2*$^{-/-}$ 2 cells on day 4 of endoderm differentiation after treatment with the two different sets of siRNAs for each during day 0-1. E) Representative phase contrast images of *NF2*$^{-/-}$ 2 cells on day 4 of endoderm differentiation. Verteporfin (VP) was applied day 0-1. Scale bars, 100 µm. F) Relative

fold-changes of *YAP1*, *TAZ,* and endoderm markers mRNA expression in *NF2*$^{-/-}$ 1 cells on day 4 of endoderm differentiation after treatment with VP day 0-1. There were no significant differences in gene expression between the VP-, DMSO-, or non-treated cells. The expression levels of the non-treated cells provided the baseline (i.e., 1). Unpaired t-tests were used for all comparisons. n.s.: $p > 0.05$, $^{*}p < 0.05$, $^{**}p < 0.01$, $^{***}p < 0.001$, $^{****}p < 0.0001$. All experiments were performed by at least 3 independent experiments with 3 replicates.

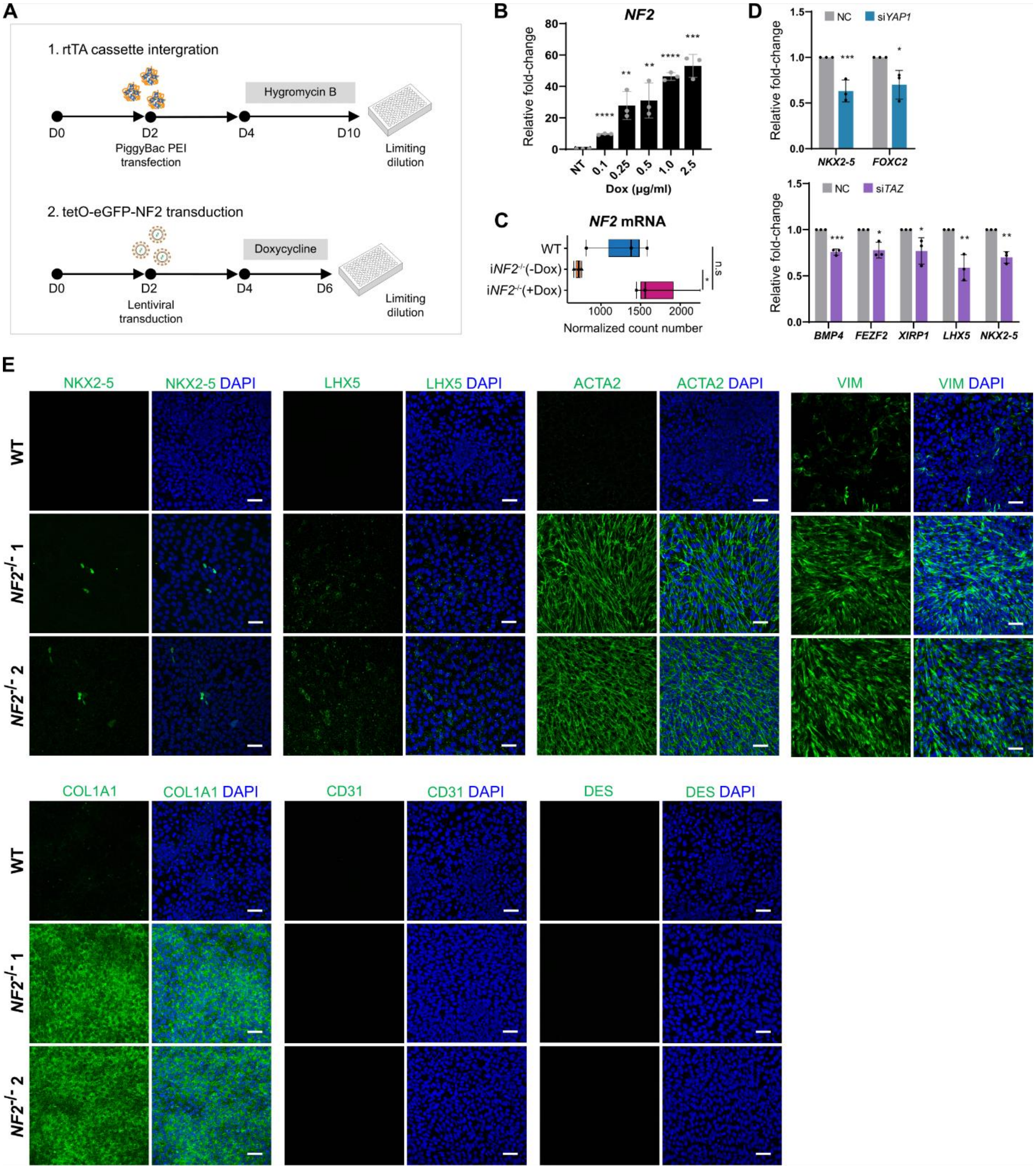

Figure S5. Identity of *NF2*$^{-/-}$ cells is myofibroblast-like under endoderm condition.

A) Schematic workflow of the establishment of Dox-inducible eGFP-NF2 transgenic hiPSC lines (i*NF2*$^{-/-}$). Genomic integrations of rtTA and Dox-inducible eGFP-NF2 cassettes were achieved by PiggyBac transposon/transposase co-transfection and lentiviral transduction, respectively. B)

Dose-response curve for Dox. RT-qPCR result showing induced *NF2* expression in i*NF2*$^{-/-}$ compared to non-treated (NT) hiPSCs. *NF2* expression in WT hiPSCs is set to 1. C) Normalized counts of *NF2* mRNA levels in WT, i*NF2*$^{-/-}$ (-Dox), and i*NF2*$^{-/-}$ (+Dox) cells under endoderm differentiation conditions that were used for RNA sequencing. D) Change in gene expression in *NF2*$^{-/-}$ 1 cells after application of *YAP1* or *TAZ* siRNA on day 4 of endoderm differentiation. When NC was set as 1, the average of *NKX2-5* and *FOXC2* expressions were 0.63 and 0.70, respectively, after treatment of si*YAP1*. Similarly, when NC was set as 1, *BMP4*, *FEZF2*, *XIRP1*, *LHX5*, *NKX2-5* expressions were 0.76, 0.78, 0.74, 0.59, 0.7, respectively, after treatment of si*TAZ*. Unpaired t-tests were used to compare expression levels. $^{*}p < 0.05$, $^{**}p < 0.01$, $^{***}p < 0.001$. E) Immunostained cells under endoderm differentiation conditions on day 7. Markers of cardiac (NKX2-5), nervous system (LHX5), myofibroblast (ACTA2, VIM, COL1A1), smooth muscle cell (CD31), and vascular endothelial (DES) development were assessed. Scale bars, 50 μm. All experiments were performed with a minimum of 3 independent experiments with 3 replicates.

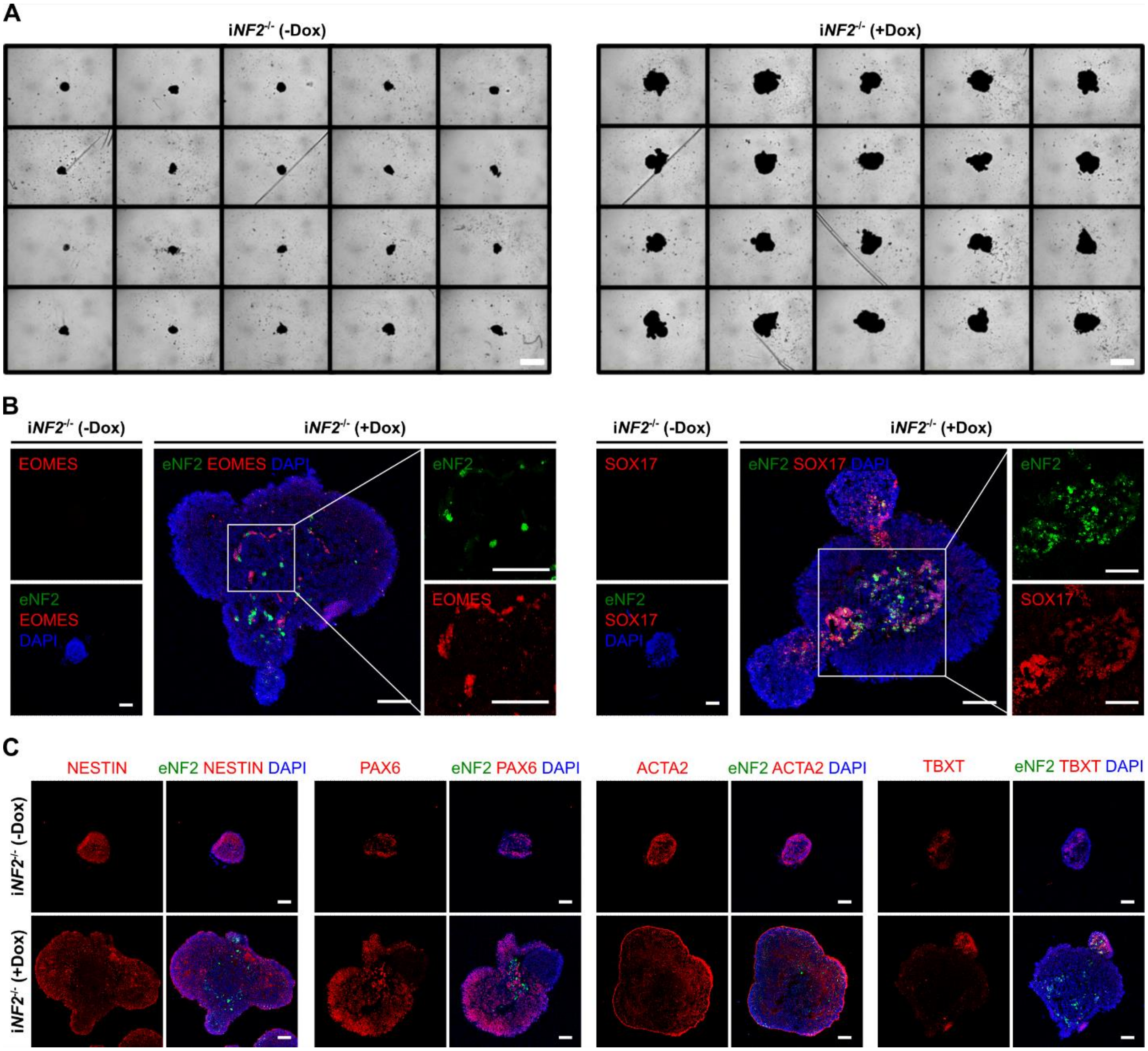

Figure S6. Characterization of doxycycline-inducible *NF2* hiPSC line.

A) Bright-field images of i*NF2*$^{-/-}$ EBs under -Dox and +Dox conditions on EB differentiation day 20. Scale bars, 800 µm. B) Immunostaining of i*NF2*$^{-/-}$ EBs (differentiation day 20) under -Dox and +Dox conditions using markers of endoderm (EOMES, SOX17). Scale bars, 100 µm. C) Immunostaining for ectoderm (NESTIN, PAX6) and mesoderm (ACTA2, TBXT) markers in i*NF2*$^{-/-}$ EBs (differentiation day 20) under -Dox and +Dox conditions. Scale bars, 100 µm. All experiments were performed with a minimum of 3 independent experiments with 2-3 technical replicates.

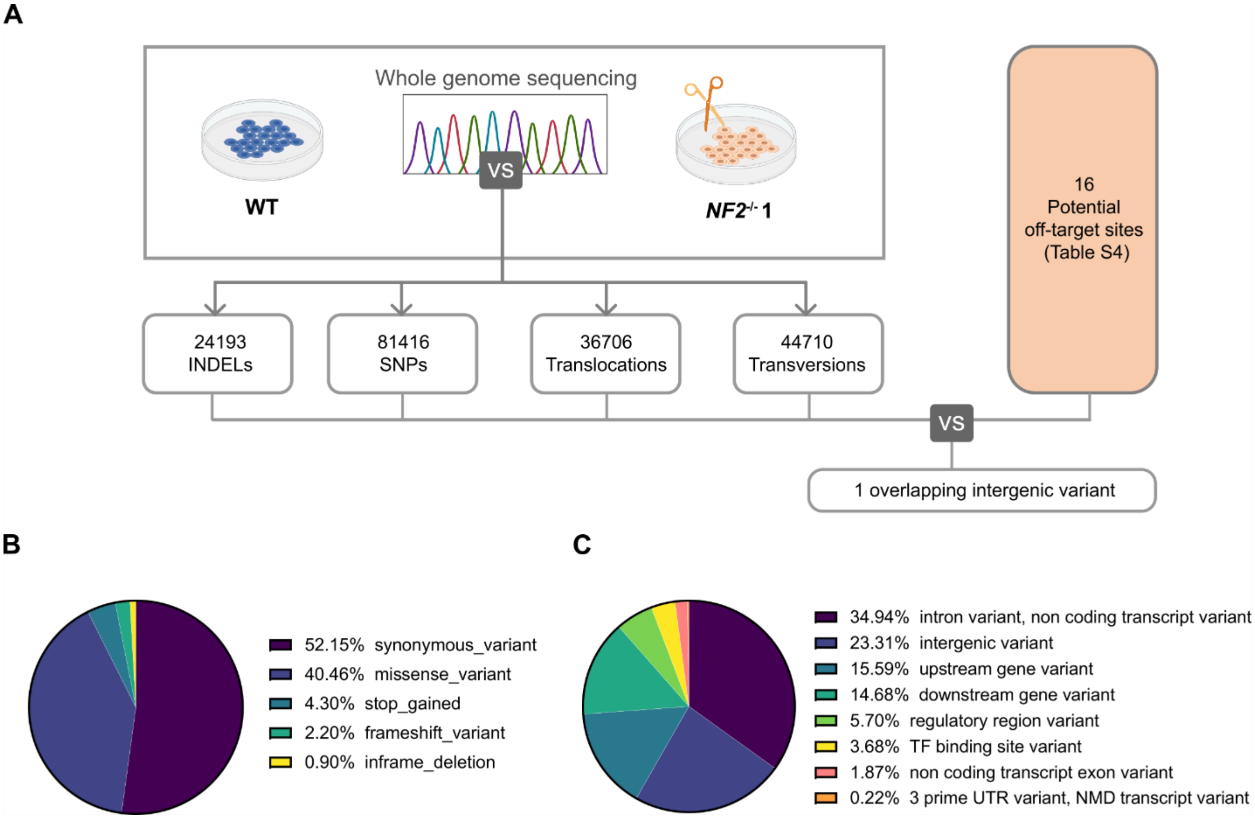

Figure S7. Somatic variant calling analysis.

A) Process chart diagrams for the variant analysis between WT and *NF2*$^{-/-}$ 1 hiPSCs. B) Types of potential mutations and their percentage in the whole genome of *NF2*$^{-/-}$ 1 hiPSCs compared with WT hiPSCs. C) Types of potential mutations and their percentage in a coding sequence of *NF2*$^{-/-}$ 1 hiPSCs compared with WT hiPSCs. Abbreviations: INDELs, insertions and deletions; SNPs, single nucleotide polymorphisms; TF, transcription factor; UTR, untranslated region; NMD, nonsense-mediated mRNA decay.

Supplemental Table S1. Previous findings related to Hippo pathway

Supplemental Table S2. RNA-seq

Supplemental Table S3. Oligo sequence

Supplemental Table S4. Somatic variant calling analysis

Supplemental Table S5. NF2 Endoderm RNA-seq sample quality control

Supplemental Table S6. Antibodies

Supplemental Table S7. Semi-quantitative image data
